# Discovery of a non‐nucleotide stimulator of interferon genes (STING) agonist with systemic antitumor effect

**DOI:** 10.1002/mco2.70001

**Published:** 2024-12-20

**Authors:** Xiyuan Wang, Zhengsheng Zhan, Zhen Wang, Yan Zhang, Kaiyan Zhao, Han Li, Xiaoqian Zhou, Yuting Guo, Fengying Fan, Jian Ding, Meiyu Geng, Xuekui Yu, Wenhu Duan, Zuoquan Xie

**Affiliations:** ^1^ State Key Laboratory of Drug Research Shanghai Institute of Materia Medica Chinese Academy of Sciences Shanghai China; ^2^ Small‐Molecule Drug Research Center Shanghai Institute of Materia Medica Chinese Academy of Sciences Shanghai China; ^3^ University of Chinese Academy of Sciences Beijing China; ^4^ Cryo‐Electron Microscopy Research Center & The CAS Key Laboratory of Receptor Research Shanghai Institute of Materia Medica Chinese Academy of Sciences Shanghai China; ^5^ Lingang Laboratory Shanghai China; ^6^ Shandong Laboratory of Yantai Drug Discovery Bohai Rim Advanced Research Institute for Drug Discovery Yantai Shandong China

**Keywords:** antitumor, immune memory, innate immunity, STING agonist

## Abstract

Agonists of the stimulator of interferon genes (STING) pathway are increasingly being recognized as a promising new approach in the treatment of cancer. Although progress in clinical trials for STING agonists in antitumor applications has been slow, there is still an urgent need for developing new potent STING agonists with versatile potential applications. Herein, we developed and identified a non‐nucleotide STING agonist called DW18343. DW18343 showed robust activation across different STING isoforms. Crystallography analysis revealed that DW18343 binds more deeply into the ligand binding domain (LBD) pocket of STING‐H232 compared to other agonists such as MSA‐2, SR‐717, or cGAMP, which likely contributes to its high potency. DW18343 triggered downstream p‐TBK1/p‐IRF3 signaling, leading to the production of multiple cytokines. Additionally, DW18343 displayed broad and long‐lasting antitumor effects in various syngeneic mouse tumor models, whether administered locally or systemically. Moreover, DW18343 induced immune memory to combat the growth of rechallenged tumors. Finally, DW18343 was shown to be an activator of both the innate and adaptive antitumor immunity in tumor tissue, potentially explaining its strong antitumor effects in vivo. In conclusion, DW18343 serves as a novel non‐nucleotide STING agonist with systemic antitumor effect through the activation of antitumor immunity.

## Graphical abstract

DW18343 specifically binds to various STING isoforms and activates downstream p‐TBK1/p‐IRF3 signaling.

DW18343 binds more deeply into a pocket formed by the ligand binding domain (LBD) of STING‐H232 as compared to other agonists such as MSA‐2, SR‐717, or cGAMP.

DW18343 demonstrated durable antitumor activity in syngeneic mouse tumor models and induced immune memory, which is associated with the activation of antitumor immunity

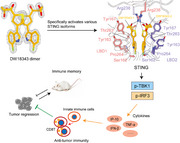

## 1 INTRODUCTION

Since the beginning of this century, the field of cancer treatment has undergone substantial transformations, resulting in marked improvements in patient outcomes. Transitioning from traditional cytotoxic drugs to targeted therapies and the rapid emergence of immunotherapies such as immune checkpoint inhibitors and chimeric antigen receptor T (CAR‐T) cell therapy highlight this progress.^1^ However, only a small subset of patients benefits significantly,^2^, ^3^ underscoring the urgent need for innovative cancer immunotherapies. Innate immunity plays a vital role in tumor surveillance and is currently the focus of intense research due to its ability to combat “cold tumors” and trigger enduring antitumor responses through immune memory.^4^ Within this context, the stimulator of interferon genes (STING) protein is being recognized as a potentially impactful target in cancer immunotherapy.^5^, ^6^

STING is vital for the functioning of both the innate and adaptive immune responses.^6^, ^7^, ^8^ When located within the intracellular endoplasmic reticulum (ER), STING undergoes structural changes and moves to the Golgi apparatus upon receiving its natural ligand, 2′3′cGMP‐AMP (cGAMP), which is synthesized by its upstream sensor, cyclic GMP‐AMP synthase (cGAS).^9^ Subsequently, it recruits and triggers the phosphorylation of TANK‐binding kinase 1 (TBK1)/interferon regulatory factor 3 (IRF3) and inhibitor of nuclear factor kappa‐B kinase (IKK)/nuclear factor kappa‐B (NF‐kB), leading to the induction of type I interferons (IFN) and various proinflammatory cytokines.^10^, ^11^, ^12^ Type I interferons can promote the tumor‐specific antigen presentation and activate tumor‐specific CD8 T cells, thereby enhancing the immune‐mediated elimination of tumor cells.^13^, ^14^, ^15^ STING also serves an immune surveillance role in tumors,^16^ as cGAMP derived from tumors can activate the STING cascade in immune cells, further bolstering the antitumor capabilities of nature killer (NK) cells.^17^ Additionally, STING activation is also involved in lysosomal‐associated cell death pathways through NACHT, LRR and PYD domains‐containing protein 3 (NLRP3)^18^ and promotes autophagy to eliminate cytoplasmic deoxyribonucleic acid (DNA) and viruses.^19^ Furthermore, STING can inhibit the activity of hexokinase 2, reducing aerobic glycolysis and promoting anti‐tumor immunity in vivo.^20^ Consequently, STING's vital role in immune surveillance and its wide array of biological functions make it an attractive candidate for cancer immunotherapy.

Agonists targeting STING have demonstrated potential in animal studies for treating tumors by converting “cold tumors” to “hot tumors”. This transformation improves the efficacy of programmed cell death protein 1 (PD‐1)/programmed cell death 1 ligand 1 (PD‐L1) immune checkpoint inhibitors^21^, ^22^, ^23^ and reveals additive antitumor effects when used in combination with leukocyte surface antigen CD47 inhibitors or angiogenesis inhibitors.^24^, ^25^, ^26^ Additionally, STING agonists have exhibited antiviral properties^27^, ^28^ and have been utilized as vaccine adjuvants.^29^, ^30^ In recent years, multiple studies have aimed to develop STING‐targeting agonists. Initially, CDN‐class agonists such as ADU‐S100^21^, ^22^ and MK‐1454^31^ demonstrated sustained antitumor activity when administered locally, but they suffered from low stability, rendering them unsuitable for systemic use. Consequently, subsequent efforts have resulted in the development of various STING agonists with improved stability for systemic administration, including di‐ABZI‐3,^32^ MSA‐2,^33^ SR‐717,^34^ HB3089,^10^ TAK676,^35^ and others.^36^ Furthermore, some genetically engineered bacteria that express STING ligands have exhibited potent anti‐tumor effects in immunocompetent mice.^37^, ^38^ However, it is worth noting that STING‐targeting agonists have exhibited limited antitumor effects in clinical trials.^39^, ^40^, ^41^ As a result, the development of new structural types of STING agonists, gaining a deeper understanding of the drug resistance mechanisms to STING agonists in clinical settings, and establishing well‐thought‐out clinical trial designs are all critically important for successful clinical translation of STING agonists in the future.

Herein, we developed a non‐nucleotide STING agonist known as DW18343. Our crystallography analysis has unveiled that the compound resides deeper into the pocket formed by the dimerized LBDs as compared to existing STING agonists (cGAMP, MSA‐2, SR‐717). DW18343 robustly activated both human and mouse STING, thereby facilitating the activation of downstream STING signaling pathways. In various tumor models, whether administered locally or systemically, DW18343 exhibited potent antitumor activity and instigates immune memory. This phenomenon is closely associated with the activation of innate immune cells and CD8T cells in the tumor immune microenvironment. Hence, DW18343 stands as a novel STING agonist meriting further investigation.

## 2 RESULTS

### 2.1 Structure‐activity relationship of the thiophene/furan compounds

**FIGURE 1:**
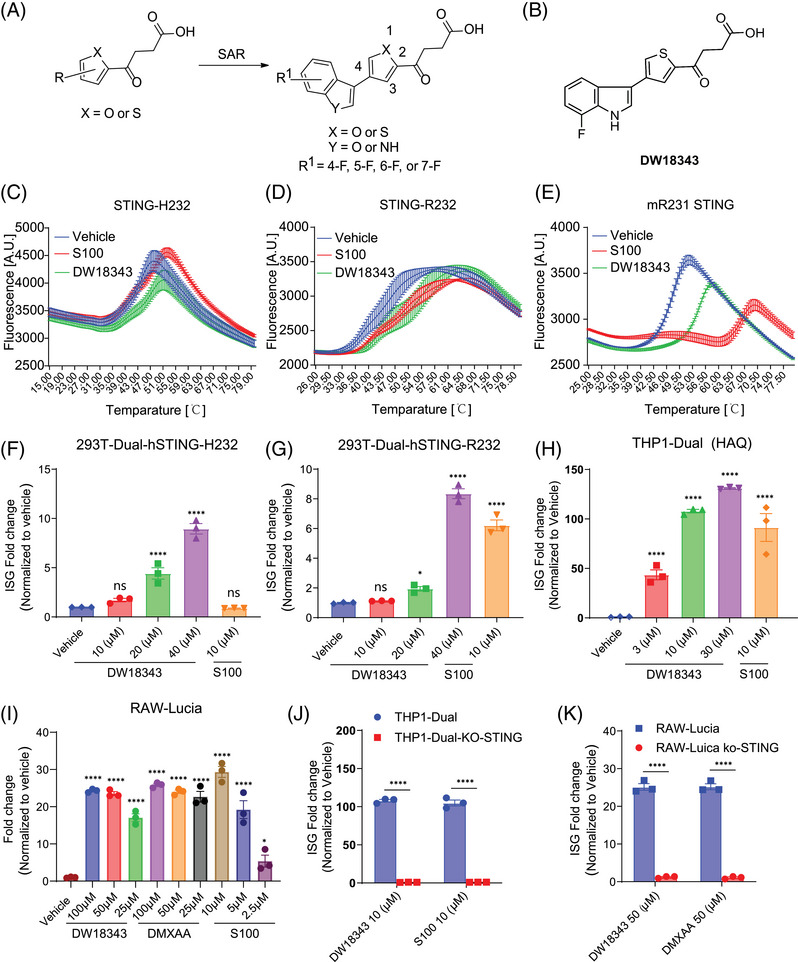
Design and evaluation of compound activity: (A) Structure activity relationship (SAR) exploration of the substituted thiophene/furan compounds. (B) Chemical structure of DW18343. (C to E) Measurement of thermal stability in the binding of compounds to human protein isoforms STING‐H232, STING‐R232, and mouse STING‐R231 using DSF, depicted as the fluorescence value. (F to I) Fold change in ISG reporter activity in 293T‐Dual‐hSTING‐H232, 293T‐Dual‐hSTING‐R232, THP1‐Dual, and RAW‐Lucia cells after 24 hours of treatment with various doses of DW18343, S100 or 5,6‐dimethylxanthenone‐4‐acetic acid (DMXAA), as compared to the vehicle. (J and K) Fold change in ISG reporter activity in THP1‐Dual and THP1‐Dual‐KO‐STING (J), RAW‐Lucia and RAW‐Lucia‐KO‐STING cells (K) after 24 hours of treatment, demonstrating the abolishment of ISG signaling activation in both THP1‐Dual‐KO‐STING and RAW‐Lucia‐KO‐STING cells. The experiments were conducted in triplicates. S100 or DMXAA were employed as positive controls. All data are presented as the mean ± SEM. Statistical difference was conducted using one‐way ANOVA. Post hoc comparisons were performed using Fisher's least significant difference (LSD) test, and the significance level was set at 95 % confidence. ANOVA, analysis of variance; STING, stimulator of interferon genes. ^*^ *p* < 0.05; ^**^ *p *< 0.01; ^***^ *p* < 0.001; ^****^ *p* < 0.0001; compared to the vehicle control (0.1%DMSO).

To evaluate the interferon‐stimulated genes (ISG)‐stimulating activities of the newly synthesized compounds, we employed THP1‐Dual cells for human ISG evaluation and RAW–Lucia cells for mouse ISG assessment, both of which feature an ISG reporter. Our initial screening for chemical inducers of ISG led us to identify the substituted thiophene/furan 4‐oxobutanoic acid species as potent ISG activators. We envisioned that incorporation of a [6 + 5] heteroaryl substituent on the 4‐position of the thiophene/furan core would enhance the STING‐agonistic activity, then we designed and synthesized a serious of thiophene/furan derivatives bearing a [6 + 5] heteroaryl group. Subsequently, we devoted significant efforts to an extensive exploration of the structure‐activity relationship (SAR), resulting in the discovery of a range of thiophene/furan derivatives featuring a 4‐position substituted benzofuran or indole, all exhibiting robust ISG‐activating capabilities (Figure 1A).

The SAR for the benzofuran or indole substituted thiophene/furan compounds were summarized in Table 1. Briefly, when the substituent is a benzofuran group, the resulting compound (DW18320) exhibited potent targeting activities for human and mouse ISG. On the other hand, among the indole‐substituted counterparts, the compound with a thiophene core displayed superior ISG‐stimulating activity compared to the one with a furan core (DW10332 vs. DW18333). DW10332 demonstrated comparable stimulation of human and mouse STING activities as the positive compounds ADU‐S100 (S100) and 5,6‐dimethylxanthenone‐4‐acetic acid (DMXAA),^42^ with changes of 87.52‐ and 21.79‐fold, respectively. However, it is worth noting that this compound underwent rapid metabolism after oral or injection administration (data not shown). To address this issue, a fluorine atom was introduced into the indole ring of DW10332 to mitigate metabolic degradation since the indole ring could not accommodate bulky substituents. Among the fluorinated compounds (DW18340, DW18342, DW18343, and DW18344), DW18343 exhibited significantly enhanced ISG‐stimulating activities, with 89.06‐ and 32.44‐fold changes in cellular human and mouse ISG signaling, respectively. As a result, DW18343 was chosen for further biological evaluation.

**TABLE 1:** Structure activity relationship (SAR) of the substituted thiophene/furan compounds on the activation of the interferon‐stimulated genes (ISG) signaling.

				ISG fold change^a^	
Compounds	X	Y	R^1^	THP1‐Dual (10 µM)^b^	RAW‐Lucia (50 µM)^b^
DW18320	O	O	H	49.52	6.98
DW10332	S	NH	H	87.52	21.79
DW18333	O	NH	H	7.01	1.87
DW18340	S	NH	6‐F	74.36	14.38
DW18342	S	NH	5‐F	44.98	1.00
DW18343	S	NH	7‐F	89.06	32.44
DW18344	S	NH	4‐F	44.57	1.03
S100	–	–	–	62.61	‐
DMXAA	–	–	–	–	36.02

Abbreviation: DMXAA, 5,6‐dimethylxanthenone‐4‐acetic acid.

^a^ All values were averaged from three independent assays.

^b^ Concentration of tested compounds.

### 2.2 DW18343 specifically targets STING to promote the activation of the ISG pathway

Given that STING plays a pivotal role as an upstream regulator of ISG signaling, we investigated whether DW18343 could directly interact with the STING protein (Figure 1B), thereby triggering the downstream ISG pathway. To this end, we utilized differential scanning fluorimetry (DSF) method^21^, ^43^ to evaluate the binding of DW18343 to various isoforms of STING proteins, employing S100 as a positive control. DW18343 and S100 were incubated with various STING isoforms, including human STING‐H232, STING‐R232, STING‐293Q, STING‐AQ, or mouse STING‐R231 in the presence of SYPRO Orange dye, and the fluorescence signals (A.U.) were measured. Our findings revealed that similar to S100, DW18343 enhanced the thermal stabilization (indicated by a right shift) of different isoforms of human STING proteins, such as STING‐H232, STING‐R232, STING‐293Q, and STING‐AQ, as well as mouse STING‐R231 protein (Figures 1C–E and S1A,B). This suggests that DW18343 can directly bind to a range of STING isoforms.

Furthermore, we examined the activating effects of DW18343 on ISG‐reporter cells harboring various isoforms of human STING protein, such as 293T‐Dual‐hSTING‐H232, 293T‐Dual‐hSTING‐R232, and THP1‐Dual (STING‐HAQ). These cells were treated with different doses of DW18343 for 24 hours, using S100 as a positive control. The results demonstrated that DW18343 dose‐dependently stimulated ISG signaling in these cells (Figure 1F–H). Moreover, we evaluated the impact of DW18343 on ISG signaling in mouse RAW–Lucia cells following 24 hours of treatment. It demonstrated activity similar to DMXAA, yet lower than S100 (Figure 1I). Besides, DW18343 demonstrated low cytotoxicity in THP‐Dual cells following 72 hours of treatment, comparable to S100 (Figure S1C).

To substantiate that DW18343 activates the ISG cascade through the STING protein, we employed STING‐knockout reporter cells, specifically THP1‐Dual‐KO‐STING and RAW‐Lucia‐KO‐STING. The results showed that DW18343 was unable to activate ISG signaling in these STING‐KO reporter cell lines (Figure 1J,K), thereby validating that DW18343 specifically targets the STING protein to activate the downstream ISG pathway.

### 2.3 Structural basis of DW18343 binding to STING

**FIGURE 2:**
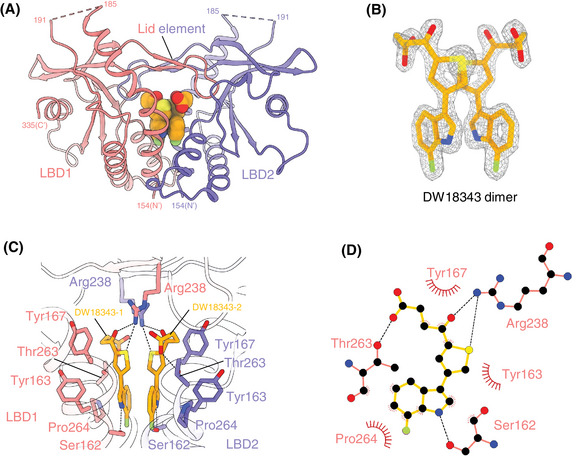
Interactions between DW18343 and stimulator of interferon genes–ligand‐binding domain (STING–LBD). (A) Overall structure of LBD in complex with DW18343. Two LBDs (red LBD1 and purple LBD2) dimerized are showed as cartoon while two molecules of DW18343 are shown as sphere. The dashed lines refer to as unmodeled residues. Four‐stranded β‐sheet forms the lid element atop DW18343. (B) 2Fo‐Fc map of DW18343 contoured at 3.0 σ. (C) Detail interaction between DW18343 and LBD. The black dashed lines refer to as the hydrogen bonds. (D) Schematic representation of the interaction between one DW18343 and its proximal LBD analyzed by LigPlot+.

Residues of LBD, excluding 186–190 and 336–341, are well‐defined (Figure 2A and Table S1). High‐quality electron density enables unambiguous modeling of DW18343 (Figure 2B). LBD dimerization forms a ligand‐binding pocket with a four‐stranded lid (Figure 2A), where two DW18343 molecules interact via pi–pi interactions of their thiophene and indole rings (Figure 2B). Each DW18343 engages its proximal LBD through hydrophobic and polar interactions (Figure 2C,D). Specifically, the ketone group, thiophene ring, and carboxyl group of each DW18343 form hydrogen bonds with Arg238 and Thr263 of its proximal LBD, respectively; the hydrophobic interaction between Tyr167 and the ketone group strengthens binding; the indole ring of each DW18343 interacts with Pro264 through hydrophobic interactions and forms a hydrogen bond with Ser162 of its proximal LBD. Additionally, the carboxyl group of each DW18343 is attracted to Arg238 of its distal LBD through an ionic interaction (Figure 2C). These interactions collectively induce the closed contraction conformation of LBDs (Figure 2A), distinct from the open conformation observed with di‐GMP or diABZ (Figure S2A). Although the conformation of the DW18343‐bound LBDs resembles that with other agonists (MSA‐2, SR‐717, or cGAMP) (Figure S2B), the binding pose of DW18343 is unique (Figure S2C), residing deeper into the pocket formed by the dimerized LBDs (Figure S2D). This distinctive conformation and binding pose likely underlie the high potency of DW18343.

### 2.4 DW18343 activates the STING downstream signaling pathway

**FIGURE 3:**
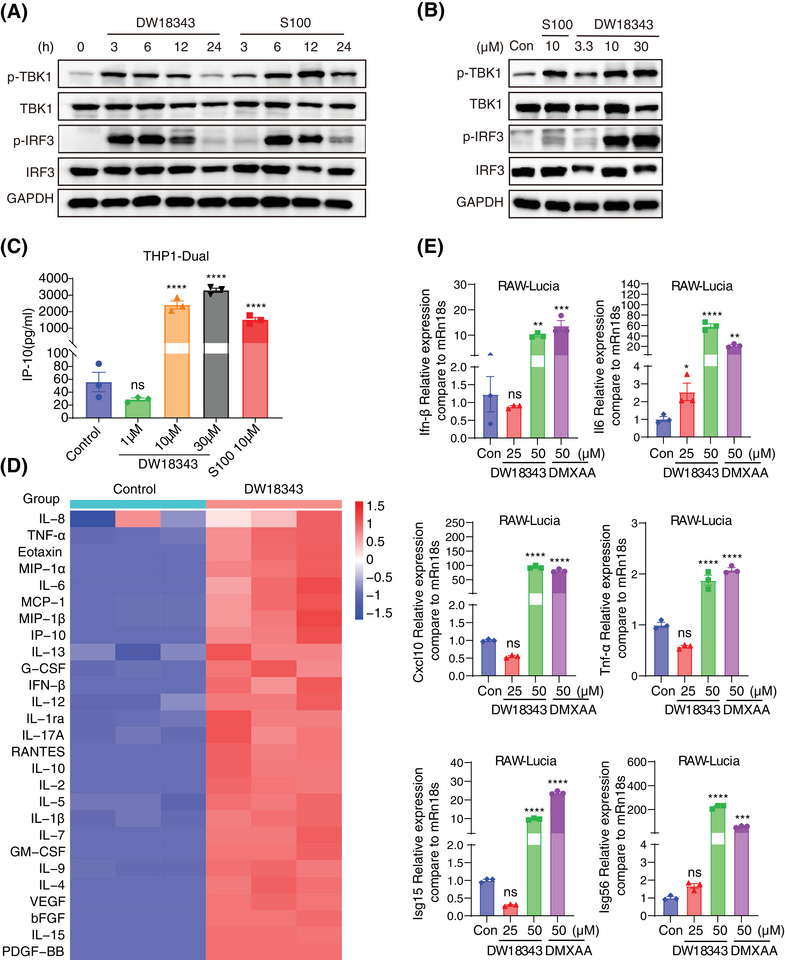
Activation of stimulator of interferon genes (STING) downstream signaling pathways. (A) THP1‐Dual cells were treated with DW18343 (10 µM) or S100 (10 µM) under indicated time points, and the expression of STING downstream TBK1/IRF3 phosphorylation pathway was determined by Western blot analysis. (B) THP1‐Dual cells were treated with indicated doses of DW18343 or S100 for 4 h, and the expression of STING downstream TBK1/IRF3 phosphorylation pathway was determined by Western blot analysis. (C) THP1‐Dual cells were treated with various doses of DW18343 or S100 for 24 h, and the concentration of IP‐10 in culture supernatant was measured by ELISA, shown as the mean ± SEM, n = 3. (D) THP1‐Dual cells were treated with DW18343 (10 µM) or vehicle control (0.1% DMSO) for 24 h and the concentrations of cytokines in the culture supernatant were detected by the Human Cytokine Antibody Array. Significantly differentially expressed cytokines are depicted in the heatmap, *n* = 3. The expression levels of cytokines were visualized using an online cluster heatmap tool (https://www.bioinformatics.com.cn). (E) RAW‐Lucia cells were treated with indicated doses of DW18343 for 6 h. The mRNA expression of interferon beta (Ifn‐β), interleukin (Il)‐6, Cxcl‐10, tumor necrosis factor alpha (Tnf‐α), interferon‐stimulated gene product 15 (Isg15) and Isg56 was determined by real‐time polymerase chain reaction (RT‐PCR), and their expression was calibrated with the internal reference mRn18s, respectively, shown as the mean ± standard error of the mean (SEM). Statistical difference was conducted using one‐way ANOVA. Post hoc comparisons were performed using Dunnett test, and the significance level was set at 95 % confidence. ^*^ *p *< 0.05, ^**^ *p *< 0.01; ^***^ *p *< 0.001; ^****^ *p *< 0.001. The experiments were carried out in triplicates.

Moving forward, we investigated the influence of DW18343 on the phosphorylation of the downstream molecules TBK1 and IRF3. THP1‐Dual cells were treated with either DW18343 or S100 for varied time points and doses, following which the phosphorylation levels of TBK1 and IRF3 were analyzed using Western blotting. Our findings showed that DW18343 time‐dependently promoted the phosphorylation of TBK1 and IRF3, with an earlier onset of activation compared to S100 (Figures 3A and S3A). Additionally, after 4 hours of treatment, DW18343 was observed to augment the phosphorylation of TBK1 and IRF3 in a dose‐dependent manner (Figures 3B and S3B). Additionally, we delved into the effect of DW18343 on the production of downstream cytokines. Initially, we focused on IP‐10 (CXCL10), a pivotal cytokine in the interferon pathway, and observed a substantial increase in IP‐10 production above the concentration of 10 µM following 24 hours of treatment, showing comparable activity to that of S100 (Figure 3C). Subsequently, we examined the impact of DW18343 on various cytokines. THP1‐Dual cells were treated with DW18343 for 24 hours and the supernatants were analyzed using a cytokine array. Interestingly, we found that DW18343 stimulated the production of a wide variety of cytokines, such as tumor necrosis factor alpha (TNF‐α), interferon beta (IFN‐β), interleukins (IL‐1β, IL‐1ra, IL‐2, IL‐4, IL‐5, IL‐6, IL‐7, IL‐8, IL‐9, IL‐10, IL‐12, IL‐13, IL‐15, IL‐17A), chemokines (eotaxin, macrophage inflammatory protein 1‐alpha [MIP‐1α], macrophage inflammatory protein 1‐beta (MIP‐1β), monocyte chemoattractant protein 1 [MCP‐1], IP‐10, RANTES/CCL5), and growth factors (granulocyte colony‐stimulating factor [G‐CSF], granulocyte‐macrophage colony‐stimulating factor [GM‐CSF], vascular endothelial growth factor [VEGF], basic fibroblast growth factor [bFGF], platelet‐derived growth factor subunit B [PDGF‐BB]) (Figure 3D). These findings indicate that DW18343 possesses potent activity in stimulating antitumor immune responses and inflammation as well as facilitating tissue damage and repair, aligning with the known functions of these cytokines.

Furthermore, we evaluated DW18343's impact on the gene expression of cytokines in mouse‐derived RAW‐Lucia cells, using DMXAA as a positive control.^42^ Following 6 hours of treatment, DW18343 was observed to enhance the gene expression of several cytokines, including Ifn‐β, Il‐6, C‐X‐C motif chemokine 10 (Cxcl‐10), Tnf‐α, IFN‐induced 15 kDa protein (Isg15), and interferon‐induced 56 kDa protein (Isg56) (Figure 3E). These results suggest that DW18343 can potentially enhance immune activation in mouse‐derived macrophages.

### 2.5 DW18343 exhibits broad‐spectrum antitumor effect

**FIGURE 4:**
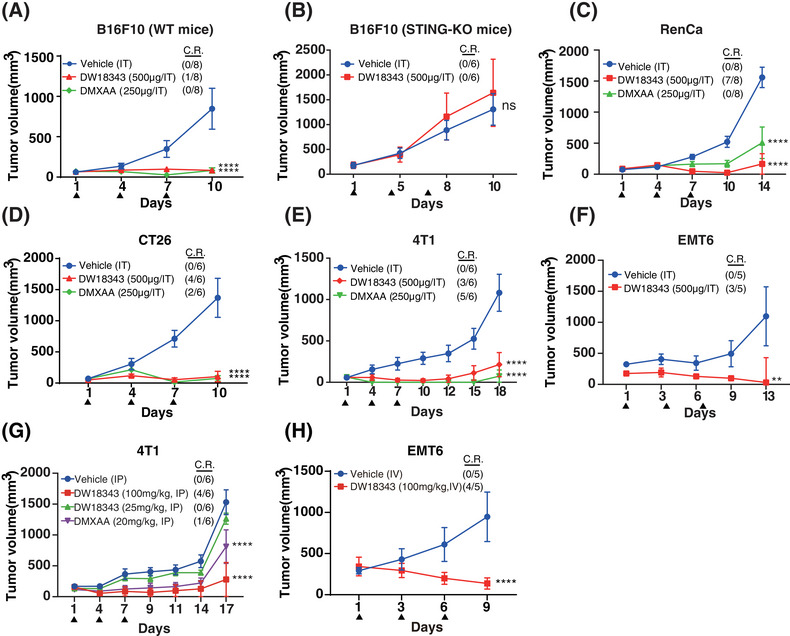
Antitumor activity of DW18343 in syngeneic tumor models. (A and B) Antitumor activity of DW18343 on B16F10 tumors in C57BL/6 WT and KO‐STING C57BL/6 mice, administered via intratumoral (IT) injections on days 1, 4, and 7. (C to F) Antitumor activity of DW18343 in RenCa, CT26, 4T1, and EMT6 tumors in Balb/c mice via intratumoral (IT) injections on days 1, 4, and 7. (G) Antitumor activity of DW18343 in 4T1 tumors in mice via intraperitoneal (IP) injections on days 1, 4, and 7. (H) Antitumor activity of DW18343 in EMT6 tumors in mice via intravenous (IV) injections on days 1, 4, and 7. DMXAA was used as a positive control, and 40% PEG400 was used as the vehicle control. The number of complete responder (C.R) mice and total mice are indicated. The Dunnett test was utilized in a two‐way analysis of variance (ANOVA) to determine differences between the treated groups and the vehicle control. Results were labeled as follows for the final day: ns, no significant difference; STING, stimulator of interferon genes; ^*^ *p* < 0.05, ^**^ *p* < 0.01; ^***^ *p* < 0.001; ^****^ *p *< 0.0001.

Considering that DW18343 markedly activated the downstream pathways of STING, we proceeded to explore its antitumor effects in immunocompetent mice. Initially, we established a syngeneic B16F10 melanoma model in C57BL/6 mice and administered DW18343 intratumorally on days 1, 4, and 7. We observed that DW18343 effectively suppressed melanoma tumor growth in vivo, with one mouse achieving complete regression. The degree of tumor inhibition was comparable to the positive control, DMXAA (Figure 4A). Importantly, this occurred without any adverse effects on mice's weight during DW18343 treatment (Figure S4A). To ascertain whether DW18343's antitumor effect operates through activating the STING pathway in vivo, we employed KO‐STING C57BL/6 mice. The results revealed that DW18343 failed to inhibit the melanoma growth in KO‐STING C57BL/6 mice (Figure 4B), and it had no impact on their body weight (Figure S4B). These findings indicate that DW18343's antitumor activity in vivo is achieved through the activation of the STING protein.

Furthermore, we conducted tests to assess DW18343's antitumor activity in immunocompetent mice across various tumor models. The results demonstrated that intermittent intratumoral injections of DW18343 markedly inhibited the growth of a variety of tumors, including RenCa renal tumors, CT26 colon tumors, 4T1 and EMT6 breast tumors. Remarkably, in some instances, mice even experienced complete tumor regression following treatment with either DW18343 or DMXAA, across these diverse tumor models (Figure 4C–F). These findings indicate that DW18343 possesses a broad‐spectrum and durable anti‐tumor effect. It is worth noting that both DW18343 and DMXAA led to a slight reduction in mice's weight in the CT26 and 4T1 tumor models, while such reduction was not observed in both the RenCa and EMT6 tumor models (Figure S4C–F). This reduction in body weight in certain cases might be related to changes occurring after tumor inhibition.

Next, we evaluated DW18343's antitumor effects through systemic administration. Initially, we examined the pharmacokinetics of DW18343 in mice using both intravenous and subcutaneous injection routes. The results revealed that intravenous injection yielded a half‐life (T1/2) of 0.86 hours and an area under the curve (AUC) of 1.44 µg·h/mL, while subcutaneous injection resulted in a T1/2 of 0.53 hours and an AUC of 1.45 µg·h/mL, achieving a 100% bioavailability (Table S2). These findings suggest that DW18343 is well‐suited for systemic administration. Subsequently, we established breast tumor models, specifically 4T1 and EMT6, in immunocompetent Balb/c mice and administered DW18343 on days 1, 4, and 7. The results demonstrated that the intraperitoneal (IP) injection of DW18343 at a dosage of 100 mg/kg led to a significant suppression of 4T1 tumors, with two‐thirds of the mice experiencing complete tumor regression. While at a dose of 25 mg/kg of DW18343 did not produce any significant tumor inhibition (Figure 4G). Additionally, there was no substantial influence on mice's weight (Figure S4G). In the EMT6 tumor model, intravenous (IV) injection of DW18343 markedly curtailed tumor growth, with 4 out of 5 tumor‐bearing mice achieving complete regression (Figure 4H). However, the mice's weight after DW18343 treatment was marginally less in comparison to the control group (Figure S4H). This variation in body weight might be associated with changes occurring as a result of tumor inhibition.

### 2.6 DW18343 stimulates the development of antitumor immune memory in mice

**FIGURE 5:**
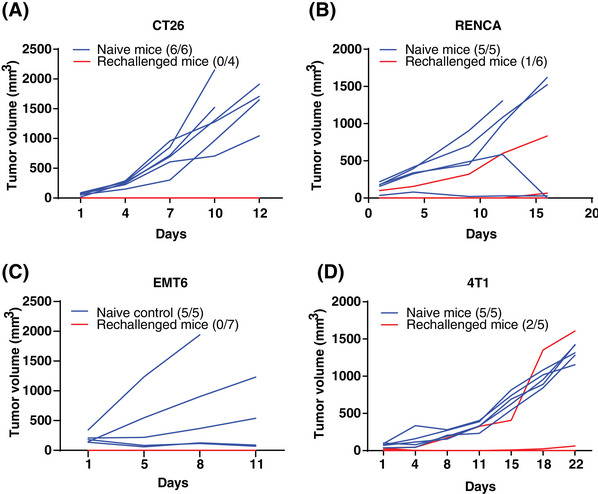
Tumor growth in naive and DW18343‐cured mice. (A to D) Growth of CT26 (A), RenCa (B), EMT6 (C), and 4T1 (D) tumors in naive mice and DW18343‐cured mice (rechallenged mice), with tumor volume measurements for each mouse. The number of tumor‐bearing mice and total mice is indicated.

As the stimulation of STING triggers innate immunity and subsequent CD8 T cell‐associated antitumor immune response, it results in establishing antitumor immune memory.^21^ Thus, we conducted tests for immune memory in mice whose tumors had regressed following DW18343 treatment. These included mice that had previously been implanted with CT26, RenCa, EMT6, and 4T1 tumor models (Figure 4C–H). Remarkably, these mice exhibited no tumor recurrence even after 2 months of DW18343 treatment. Then, we rechallenged these mice with the corresponding tumor cells and then observed tumor growth. We compared the results to age‐matched controls. In the CT26 tumor model, all four mice previously cured by DW18343 developed immune memory, effectively resisting the growth of re‐implanted tumors, while all six naive mice developed tumors (Figure 5A). In the RenCa tumor model, among the six mice previously cured by DW18343, one exhibited tumor growth while the other five successfully resisted tumor growth. In contrast, all naive mice developed tumors (Figure 5B). In the EMT6 tumor model, all seven mice previously cured by DW18343 developed immune memory and effectively resisted tumor reimplantation (Figure 5C). Furthermore, in the 4T1 tumor model, among the five mice previously cured by DW18343, one experienced faster tumor growth, one had slower growth, and the remaining three showed resistance to tumor growth (Figure 5D). Collectively, most mice that were cured by DW18343 developed immune memory, demonstrating effective resistance to the growth of re‐implanted tumor cells in vivo. This underscores DW18343's capability to effectively induce the formation of antitumor memory immune cells.

### 2.7 DW18343 triggers the antitumor immunity

As previously mentioned, the induction of antitumor immune memory via DW18343 depends on its ability to stimulate the host's immune system. To further explore this, we performed a comprehensive assessment of DW18343's effects on immune cell subsets within the tumor microenvironment. We administered DW18343 intratumorally into RenCa tumors for 72 hours and subsequently collected tumor tissue samples. We utilized flow cytometry to assess the proportions of different immune cell subsets within the tumors.

**FIGURE 6:**
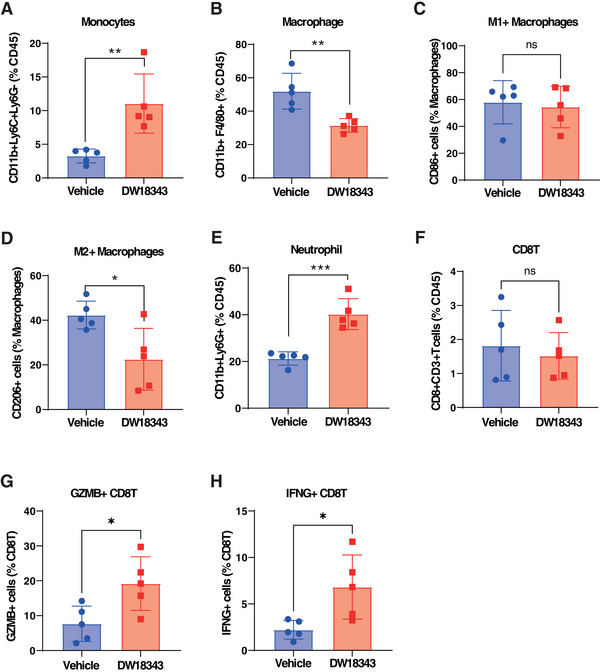
Alterations in immune cell subpopulations in tumor tissues during DW18343 treatment. (A to H) The RenCa tumor‐bearing mice were intratumorally treated with 40% PEG400 or DW18343 (500 µg) for 72 h (*n* = 5). Tumor tissues were isolated, and the proportion of monocytes, macrophages, M1‐macrophages, M2‐macrophages, neutrophils, CD8T, GZMB^+^CD8T, and IFNG^+^CD8T cells was analyzed by flow cytometry. Student *t*‐test was performed to determine differences between groups. ^*^ *p *< 0.05, ^**^ *p *< 0.01, ^***^ *p *< 0.001.

Concerning innate immune cells, we observed that DW18343 could augment the proportion of monocytes while reducing the proportion of macrophages (Figure 6A,B). However, there was no significant effect on the proportions of dendritic cells (DC) and natural killer (NK) cells (Figure S5A,B). Furthermore, when we performed an analysis of macrophage subtypes, DW18343 had no notable impact on M1‐like macrophages but did reduce the proportion of immunosuppressive M2‐like macrophages (Figure 6C,D). Additionally, DW18343 increased the proportion of neutrophils (Figure 6E). These findings indicate that DW18343 exerts its antitumor effects by activating the innate immunity.

Moving on, we conducted an analysis of the proportions of adaptive immune cells and observed that DW18343 resulted in a decrease in the proportion of CD4T cells (Figure S5C). However, it did not significantly influence the proportion of CD8T cells (Figure 6F). Despite the unchanged proportion of CD8T cells, DW18343 notably increased the proportion of antitumor CD8T cell subsets, including granzyme B (GZMB^+^)‐CD8T cells and interferon‐γ (IFNG^+^)‐CD8T cells (Figure 6G,H). These findings suggest that DW18343 enhances the activation of CD8T cells' antitumor capabilities, which could potentially contribute to the development of an antitumor immune memory, possibly through the differentiation of some cells into memory CD8T cells.

Additionally, we examined the effects of DW18343 on subpopulations of immune cells isolated from mouse peripheral blood. These cells were treated with DW18343 for 72 hours and then subjected to flow cytometric analysis. Our findings indicated an increase in monocytes, macrophages, neutrophils, and GZMB^+^CD8T, along with a reduction in M2‐macrophages, besides there were no significant changes observed in M1‐macrophages, CD8T, and IFNG^+^CD8T cells (Figure S6A–H). This data corroborates the stimulatory influence of DW18343 on an array of immune cell subtypes.

## 3 DISCUSSION

In the current study, we unveil a novel non‐nucleotide STING agonist named DW18343, shedding light on its chemical structure and biological functions. DW18343 possesses the capability to bind to and activate various forms of human STING as well as mouse STING proteins, thereby facilitating the initiation of the ISG pathway. In the context of human and mouse STING‐KO reporter cells, the activation of the ISG pathway was abolished, confirming its precise targeting of the STING protein to trigger the ISG pathway. Upon conducting a crystal structure analysis, we made a revealing discovery. While DW18343, like established STING agonists such as cGAMP, MSA‐2, and SR‐717, binds to the LBD region of the STING protein, it stands out due to its side chain, which delves even deeper into the LBD region of the human STING‐H232 subtype. This unique binding characteristic clarifies why DW18343 demonstrates more robust activation of the STING‐H232 subtype when compared to S100. Consequently, DW18343's capacity to activate multiple STING isoforms underscores its potential applicability across a broad spectrum of patient populations.

DW18343 demonstrates a dose‐ and time‐dependent activation of TBK1 and IRF3 phosphorylation downstream of STING. It also notably enhances the production of several cytokines associated with anti‐tumor activity, including IFN‐β, IL‐2, IL‐12, and IP‐10, among others. Specifically, IFN‐β plays crucial roles in activating innate immunity and facilitating antigen presentation, thereby promoting the activation of CD8T cells in antitumor responses.^14^, ^44^, ^45^ IL‐2 is known for its ability to stimulate the differentiation of T cells and NK cells, both of which are key players in antitumor responses.^46^ IL‐12, on the other hand, activates Th1 cells, NK cells, and cytotoxic T cells, which is crucial for antitumor immunity.^47^ IP‐10 (CXCL10) regulates the chemotaxis of T cells to the tumor site, enhancing their differentiation and activation, thus exerting anti‐tumor effects.^48^ Therefore, the increased expression of these cytokines likely mediates the antitumor effects of DW18343. In addition, our research revealed an elevation in various inflammatory cytokines, including TNF‐α, IL‐1β, and IL‐6, as well as immunosuppressive cytokines like IL‐10, indicating that DW18343 has the potential to induce substantial inflammation. Furthermore, DW18343 promotes the production of chemokines such as eotaxin, MIP‐1a, MIP‐1b, MCP‐1, and RANTES, along with growth factors including G‐CSF, GM‐CSF, VEGF, bFGF, and PDGF‐BB. This suggests an increase in immune activation and tissue repair functions. Consequently, DW18343 may have a wide range of biological effects, but it is important to note that the increase in some cytokines may also lead to cytokine storms. Further research is necessary to explore whether these cytokines can serve as toxicity biomarkers for STING agonists. Furthermore, in mouse‐derived macrophages, DW18343 also stimulated the expression of several cytokines, including Ifn‐β, Il‐6, Cxcl‐10, Tnf‐α, Isg15, and IsgG56, underscoring its capability to simulate the STING axis in mice.

In syngeneic tumor models, DW18343 has showcased potent antitumor efficacy against various tumor types, encompassing melanoma, renal tumors, colon tumors, and breast tumors. Furthermore, it is worth noting that the antitumor effect of DW18343 diminishes in STING‐KO mice, underscoring that its antitumor impact is mediated through the STING protein in vivo. DW18343 also exhibits favorable pharmacokinetic properties and impressive antitumor activity when administered intraperitoneally or intravenously. This expands the range of administration routes and applicable tumor models, making it a versatile candidate for cancer therapy. DW18343 exhibits higher stability compared to cyclic dinucleotide (CDN) STING agonists, which are readily metabolized by cytoplasmic and circulating enzymes like phosphodiesterase.^49^ Unlike the case with antibody‐drug conjugates (ADCs) that incorporate a STING agonist,^50^, ^51^ where continuous activation of the cGAS‐STING pathway could potentially cause severe toxicity.^51^ Additionally, DW18343 has demonstrated the ability to induce immune memory. This is evident when these mice are subsequently exposed to the same tumors; their immune systems spring into action to prevent tumor recurrence. This finding aligns with prior reports^22^, ^35^, ^43^ and underscores the potential of DW18343 in bolstering long‐lasting immune responses against tumors.

Furthermore, we conducted an in‐depth analysis of DW18343's impact on the tumor microenvironment to delve into its immunological mechanisms against tumor growth. In the RenCa tumor model, we observed that DW18343 led to an increase in the proportion of monocytes and neutrophils, simultaneously reducing the immunosuppressive M2‐like macrophages. This orchestration serves to prime the anti‐tumor capabilities of innate immune cells.^52^, ^53^, ^54^ Nevertheless, the proportions of DCs and NK cells remained relatively stable, though their functional activation could be enhanced. Regarding to the adaptive immune cells, DW18343 was found to decrease the proportion of CD4T cells, likely attributable to cell death triggered by excessive activation.^55^ However, DW18343 did not significantly affect the proportion of CD8T cells. Interestingly, DW18343 did bring about a noteworthy increase in the GMZB^+^CD8T and IFNG^+^CD8T cell subsets, both of which play pivotal roles as effector cells in antitumor immunity.^10^, ^22^ Consistent with the in vivo results, we found that DW18343 also increased the monocytes, neutrophils, GMZB^+^CD8T and decreased the M2‐macrophage in vitro. While there was an increase in macrophage that was different from in vivo result, which reflects the immunostimulatory effect of DW18343 in blood. It is essential to bear in mind that alterations in immune cell populations can be affected by multiple factors, including tumor type, treatment duration, and administration dosage. Nevertheless, the underlying consistency lies in the fact that STING agonists, like DW18343, promote the activation of both the innate and adaptive antitumor immunity, with the ability for a portion of activated T cells to differentiate into memory T cells.^10^, ^21^, ^22^, ^34^

Meanwhile, our study has several limitations. Firstly, while we have investigated the impact of DW18343 on the subtypes of immune cells within both the tumor microenvironment and in vitro settings, our comprehension of the intricate immunological mechanisms underlying these effects remains incomplete, especially concerning the variable responses of immune cell subtypes across different tumor cell types and treatment durations. Secondly, we followed the commonly used literature protocol of administering the treatment on days 1, 4, and 7 to observe the antitumor effects; however, the optimal administration method to achieve the best antitumor effects remains unclear and requires further exploration. Thirdly, our antitumor activity tests in vivo were conducted with monotherapy, and the potential for combination with other drugs was not explored and warrants further investigation.

In summary, this study introduces a novel STING agonist, DW18343, with the capacity to activate different isoforms of human and mouse STING proteins. Notably, crystal structure analysis reveals that DW18343 resides deeper into the pocket formed by the dimerized LBDs binding domain (LBD), likely underlying its high potency. DW18343 precisely triggers the STING pathway, propelling the phosphorylation of TBK1 and IRF3, as well as the release of downstream interferon pathways and various cytokines. This activation, in turn, amplifies the antitumor capabilities of both innate and adaptive immune cells. Interestingly, DW18343 exhibits favorable pharmacokinetic properties and demonstrates remarkable efficacy in curbing the growth of diverse tumors when administered either locally or systemically. It has the potential to lead to complete regression of tumors and elicits enduring immune memory effects in mice. Consequently, DW18343 offers a novel option for the clinical development of STING agonists.

## 4 MATERIALS AND METHODS

### 4.1 Protein expression and purification

The expression and purification of STING proteins were carried out as described previously.^10^ Briefly, the ligand binding domain (LBD, residues 154–341) from human STING‐H232 was inserted into a modified pET‐M3C vector, resulting in a N‐terminal His6‐tagged protein followed by a PreScission protease site. *Escherichia coli* BL21 (DE3) strain was employed to express the recombinant protein. After incubation at 20°C for 16 hours, the cells were harvested and resuspended in the binding buffer (300 mM NaCl, 10 mM imidazole, and 20 mM Tris pH 8.0). Then the cells were sonicated, and the lysate was centrifugated at 100,000 g for 25 min. The resulting supernatant was treated with Ni^2+^‐NTA agarose (Smart‐Lifesciences), and the bound protein was eluted with binding buffer containing 250 mM imidazole. Further purification was achieved using size‐exclusion chromatography (SEC) with the SEC buffer (20 mM Tris pH 8.0 and 100 mM NaCl). For crystallization, the N‐terminal tag of the recombinant protein was cleaved with homemade PreScission protease and further purified by SEC.

### 4.2 Crystallography

Purified LBD protein was concentrated to about 10 mg/mL and incubated with excessive DW18343 for crystallization. Crystals were obtained via a hanging‐drop vapor diffusion method in a reservoir solution of 20% w/v polyethylene glycol monomethyl ether 5,000 and 0.1 M Bis‐Tris pH 6.5, at 20 °C. The crystals were kept in a crystallization solution containing 25% glycerol and flash‐frozen. X‐ray diffraction data were collected at the Shanghai Synchrotron Radiation Facility, beamline BL17U1 (0.9792 Å), and processed using HKL2000.^10^, ^56^ The complex structure was solved by molecular replacement using the structure of STING LBD bound with MSA‐2 (PDB ID: 6UKM^33^) as the search model.^10^ The model was rebuilt and adjusted with Coot,^57^ and refined by the phenix.refine program of PHENIX.^58^ According to the Ramachandran plot, 97.11% residues of the final model were located in the favored region without outliers. The complete refinement statistics are shown in Table S1. The interaction between DW18343 and its proximal LBD was analyzed using LigPlot+.^59^ Coordinates of the crystal structure have been deposited in the Protein Data Bank under the accession code 9IJN.

### 4.3 Differential scanning fluorimetry (DSF)

The DSF assay was employed to assess the binding of the compound to the STING protein.^60^ We obtained the human STING protein (R232, H232, 293Q, AQ) bearing cytosolic residues (140‐379) and the mouse STING (R231) containing the residues (139‐378) from Novoprotein. STING protein (5 µM), DW18343 (50 µM), and SYPRO Orange protein gel stain (5000X) (Cat. code: S6651, Invitrogen) were mixed, then added an equal volume of a reaction buffer (150 mM NaCl and 10 mM HEPES, pH 7.5) to reach 40 µL as a total volume.^61^ S100 was employed as a positive control. The HEX fluorescence signal (A.U.) was measured using a CFX96 Real‐Time System (Bio‐Rad) with a temperature increase set at a scan rate of 0.5°C/min, ranging from 25°C to 80°C. This test was conducted in triplicate.

### 4.4 Cells and reagents

THP1‐Dual Cells (Cat. code: thpd‐nfis), RAW‐Lucia Cells (Cat. code: rawl‐isg), THP1‐Dual KO‐STING Cells (Cat. code: thpd‐kostg), RAW‐Lucia KO‐STING Cells (Cat. code: rawl‐kostg), 293T‐Dual hSTING‐H232 Cells (Cat. code: 293d‐h232), 293T‐Dual hSTING‐R232 Cells (Cat. code: 293d‐r232), and ADU‐S100 (Cat. Code: tlrl‐nacda2r‐01) were all sourced from InvivoGen. DMXAA (Cat. Code: 117570‐53‐3) was purchased from Meilunbio.^61^ All cells were maintained at 37°C in a humidified environment with 5% CO_2_, following the manufacturer's guidelines.^43^ The Cell RNA purification Kit (Cat. code: B0004D) and tissue RNA purification Kit Plus (Cat. code: EZB‐RN001‐PLUS) were obtained from EZB, while primers were provided by Sangon Biotech. Additionally, antibodies for phospho‐TBK1 (Ser172) (Cat. code: 5483S), TBK1 (Cat. code: 3504S), phospho‐IRF3 (Ser396) (Cat. code: 4947S), IRF3 (Cat. code: 4302S), and GAPDH (Cat. code: 5174S) were procured from Cell Signaling Technology.^10^

### 4.5 ISG reporter assay

THP1‐Dual cells, THP1‐Dual KO‐STING cells (1 × 10^5^ cells/well), 293T‐Dual hSTING‐H232 or 293T Dual hSTING‐R232 (0.8 × 10^5^ cells/well), RAW‐Lucia, and RAW‐Lucia‐KO‐STING cells (0.8 × 10^5^ cells/well) were suspended in 180 µL of medium per well in a 96‐well plate.^10^ To these, specified doses of a compound were added to achieve a final volume of 200 µL, and the cells were then incubated for 24 h.^10^ Following this incubation period, 20 µL of the supernatant combined with 50 µL of QUANTI‐Luc (Cat. code: repqlc2, Invivogen) detection reagent was transferred to a black 96‐well plate (catalog code: 3601; Corning), adhering to the provided protocol. Luminescence readings were taken using SpectraMAX plus 384 (Molecular Devices). The ISG‐fold change was calculated relative to the vehicle control.^60^

### 4.6 Human cytokine antibody array

THP1‐Dual cells were exposed to either the vehicle (0.1% DMSO) or DW18343 (10 µM) for 24 hours (*n* = 3), and the levels of cytokines in the cell supernatant were determined using the Bio‐Plex Pro Human Cytokine 27‐plex Panel (#M500KCAF0Y, Bio‐Rad) kit according to the manufacturer's instructions, performed by Wayen Biotechnologies Inc. The concentrations of the analytes in these assays were calculated using a standard curve provided by the manufacturer's software. The expression levels of cytokines that showed significant changes following DW18343 treatment compared to the vehicle (*p *< 0.05, unpaired *t*‐test) were visualized using an online cluster heatmap tool (https://www.bioinformatics.com.cn).

### 4.7 Western blotting

THP1‐Dual cells (1 × 10^6^ cells/well) were seeded in a 6‐well plate and treated with DW18343 (10 µM) for various time periods or exposed to different doses of DW18343 for 4 h, with S100 as a positive control. To collect cell lysates, 1× SDS‐PAGE Sample Loading Buffer (Cat. code: P0015, Beyotime) was employed, and samples were subsequently heated at 100°C for 30 min. These lysates were then electrophoresed on 10% SDS‐PAGE gels and transferred onto nitrocellulose (NC) membranes. These membranes were incubated with 3% BSA for an hour to block nonspecific binding, and then incubated overnight at 4°C with antibodies targeting phospho‐TBK1 (Ser172), TBK1, phospho‐IRF3 (Ser396), IRF3, and GAPDH. Following this, the membranes were incubated with horseradish peroxidase‐conjugated secondary antibodies. Detection was accomplished using an enhanced chemiluminescence (ECL Plus, Cat. code: 17050622, BioRad).^10^ Grayscale values were analyzed using ImageJ software (National Institutes of Health).

### 4.8 Enzyme‐linked immunosorbent assay (ELISA)

THP1‐Dual cells were seeded in 96‐well plates at a density of 1 × 10^5^ cells per well and exposed to specified concentrations of DW18343 for 24 hours. The levels of cytokines in the cell supernatants were measured using a human IP‐10 kit (Cat. code: 550926, BD) according to the instructions provided by the manufacturer. The concentration of IP‐10 (pg/mL) was quantified using a standard curve.

### 4.9 Real‐time PCR

RAW‐Lucia cells (5 × 10^5^/well) were plated in 12‐well plates and exposed to specified concentrations of DW18343 or DMXAA for 6 h. Total cellular RNA was extracted using the EZ‐press RNA purification kit (Cat. Code: B0004D), and cDNA was synthesized using the ABScript Neo RT Master Mix for qPCR with gDNA remover (RK20433). Real‐time PCR was conducted with 2X Universal SYBR Green Fast qPCR Mix (RK21203) obtained from Abclonal Technology. The primer sequences were as follows: Ifn‐β forward, 5′‐ AGCTCCAAGAAAGGACGAACA‐3′, Ifn‐β reverse, 5′‐ GCCCTGTAGGTGAGGTTGAT‐3′; Il‐6 forward, 5′‐GCCTTCTTGGGACTGATGCT‐3′, Il‐6 reverse, 5′‐TGTGACTCCAGCTTATCTCTTGG‐3′; Cxcl‐10 forward, 5′‐CCAAGTGCTGCCGTCATTTTC‐3′, Cxcl‐10 reverse, 5′‐GGCTCGCAGGGATGATTTCAA‐3′. Isg15 forward, 5′‐GGAACGAAAGGGGCCACAGCA‐3′, Isg15 reverse, 5′‐CCTCCATGGGCCTTCCCTCGA‐3′; Isg56 forward, 5′‐CCGTAGGAAACATCGCGTAGA‐3′, Isg56 reverse, 5′‐AGCCATGCAAACATAGGCCA‐3′; Tnf‐α forward, 5′‐TATGGCCCAGACCCTCACA‐3′, Tnf‐α reverse, 5′‐GGAGTAGACAAGGTACAACCCATC‐3′; Rn18s forward, 5′‐GCAATTATTCCCCATGAACG‐3′, Rn18s reverse, 5′‐GGCCTCACTAAACCATCCAA‐3′;

Rn18s was used as a reference control. The expression of genes (ΔΔCT) was calculated as relative to the vehicle control.^60^

### 4.10 Pharmacokinetics

Animal procedures were approved by the Institutional Animal Care and Use Committee of the Shanghai Institute of Materia Medica. CD‐1 mice weighing between 25 and 28 grams were utilized for the pharmacokinetics study, with each group consisting of three mice. The mice were housed in a controlled environment with a room temperature maintained between 18°C and 29°C and a relative humidity ranging from 30% to 70%. A 12‐hour light/12‐hour dark cycle was regulated by an electronic time‐controlled lighting system. The mice were provided with a standard rodent diet and had unrestricted access to water. Prior to administration, the mice underwent a 12‐h fasting period and continued to fast for an additional 2 h. DW18343 (5 mg/kg) was administered to the mice via intravenous (IV) or subcutaneous (SC) injection, and plasma samples were collected at various time points (0.05 h, 0.25 h, 0.75 h, 2 h, 4 h, 8 h, 24 h) to determine the concentrations of DW18343. Pharmacokinetic parameters for DW18343 were subsequently calculated.

### 4.11 Animal studies

Animal procedures were approved by the Institutional Animal Care and Use Committee of the Shanghai Institute of Materia Medica (Number: 2020‐04‐GMY‐17). 6‐week‐old Balb/c mice and C57BL/6 mice were procured from LingChang Biotechnology. C57BL/6 mice with a stable STING gene knockout (KO‐STING) were obtained from Model Organisms.^61^ A 12‐hour light/12‐hour dark cycle was regulated by an electronic time‐controlled lighting system. The mice were provided with a standard rodent diet and had unrestricted access to water. When the tumor volume of the mouse exceeds 2000 mm^3^ or the weight loss >10%, the experiment will be concluded on the same day.

For the tumor models, B16F10 cells (1 × 10^5^) were subcutaneously injected into the right armpit of C57/BL6 (n = 8) or C57/BL6 KO‐STING mice (n = 6). RenCa (5 × 10^5^), 4T1 (5 × 10^5^), CT26 cells (5 × 10^5^), and EMT6 (5 × 10^5^) were similarly injected subcutaneously into the right armpit of Balb/C mice (n = 5~8). Once the tumors reached a size of approximately 50~100 mm^3^. The mice received intermittent treatments of DW18343 via intratumoral (IT), intraperitoneal (IP), or intravenous (IV) injection at the indicated concentrations on days 1, 4, and 7. The vehicle control was administered with 40% PEG400.^43^

For the CT26, RenCa, EMT6, and 4T1 rechallenge model, mice that had completely regressed tumors following DW18343 treatment were monitored for 2 months of tumor‐free survival. Subsequently, these mice were reinoculated with the same tumor cells, while age‐matched naive mice served as control subjects. Tumor volume and body weight were recorded twice a week, with tumor volume (TV) calculated using the formula: *V = (a × b* ^2^)/2 (*a*, length; *b*, width).^43^

### 4.12 Analysis of immune cell subsets in tumor tissues

Animal procedures were approved by the Institutional Animal Care and Use Committee of the Shanghai Institute of Materia Medica. Balb/C mice were utilized to establish RenCa tumors. Once the tumor volume reached approximately 400 mm^3^, the mice received intratumoral injections of either the vehicle (40% PEG400) or DW18343 (500 µg) for 72 hours. Immunotyping was conducted following a previously established method.^26^ In brief, tumor tissues were excised, minced, and digested using the Tumor Dissociation Kit (mouse) (Cat: 130‐096‐730, Miltenyi) protocol. After one hour of shaking at 37°C, the purified single cells were obtained and filtered through a 70 µm strainer. Cell counts were determined, and the cells were plated into Corning R 96‐well Clear V‐Bottom TC treated Microplates (Cat: 3894, Corning). Next, the cells were resuspended in FACS buffer and exposed to surface antibodies for 20 min in the dark. To identify live cells, Zombie NIR Fixable Viability Kit (Cat: 423105, Biolegend) was used for a 15‐min dark incubation.^10^, ^61^ Surface antibodies included CD45 (Cat:103116, Biolegend), CD3 (Cat:11‐0031‐85, ThermoFisher), CD8 (Cat:100734, Biolegend), CD11b (Cat:564454, BD), Ly6C (Cat:128044, Biolegend), Ly6G (Cat:127616, Biolegend), CD11c (Cat: 117318, Biolegend), F4/80 (Cat:565411, BD), CD86 (Cat:564199, BD), CD206 (Cat:141729, Biolegend), and CD49b (Cat:564454, Biolegend). For intracellular protein staining, the cells were fixed, permeabilized, and stained with antibodies against intracellular molecules, including granzyme B (GZMB) (Cat: 17‐8898‐82, ThermoFisher Scientific) and IFN‐γ (Cat: 505836, Biolegend). Following staining, the samples were fixed with 1% paraformaldehyde in the dark for 15 min.^61^ After washing, each sample was resuspended in FACS buffer and analyzed using BD LSRFortessa.^61^ The raw data collected were processed using Flowjo software.

### 4.13 Statistical analysis

Statistical analyses were carried out using GraphPad Prism 10.1.2 software, utilizing one‐way ANOVA analysis or two‐way ANOVA analysis of variance with Dunnett multiple‐comparison test, or the Fisher's LSD test as appropriate.

## AUTHOR CONTRIBUTIONS

Zuoquan Xie, Wenhu Duan, Xuekui Yu, Meiyu Geng, and Jian Ding: Designed research. Zuoquan Xie, Xiyuan Wang, Yan Zhang, and Han Li: Performed biological research. Wenhu Duan, Zhengsheng Zhan, Kaiyan Zhao, Xiaoqian Zhou, and Yuting Guo: Designed and synthesized compounds. Zhen Wang, Fengying Fan, and Xuekui Yu: Performed structural analysis. Wenhu Duan, Zhengsheng Zhan, and Zuoquan Xie: Analyzed data. Jian Ding and Meiyu Geng: Provided scientific support. Xiyuan Wang, Zhengsheng Zhan, and Zhen Wang: Wrote the paper. Zuoquan Xie, Wenhu Duan, and Xuekui Yu: Revised the paper. All authors have read and approved the final manuscript.

## CONFLICT OF INTEREST STATEMENT

The authors declare no conflicts of interest.

## ETHICS STATEMENT

Animal procedures were approved by the Institutional Animal Care and Use Committee of the Shanghai Institute of Materia Medica (Number: 2020‐04‐GMY‐17).

## Supporting information



Supporting Information

## Data Availability

The data that support the findings of this study are available on request from the corresponding author.
