# Supplementary material for: Discovery of a non‐nucleotide stimulator of interferon genes (STING) agonist with systemic antitumor effect
Source: MedComm (2020). 2024 Dec 20;6(1):e70001. doi: 10.1002/mco2.70001 (PMC11661907; doi:10.1002/mco2.70001)
Supplement: Supplementary file 1 — Supporting Information [file MCO2-6-e70001-s001.docx]

**Supplementary Materials**

**Discovery of a non-nucleotide Stimulator of Interferon Genes (STING) agonist with systemic anti-tumor effect**

Xiyuan Wang^1,#^, Zhengsheng Zhan^2,3,#^, Zhen Wang^4,#^, Yan Zhang^1^, Kaiyan Zhao^2,3^, Han Li^1,5^, Xiaoqian Zhou^2,3^, Yuting Guo^2,3^, Fengying Fan^3,4^, Jian Ding^1,3,6^, Meiyu Geng^1,3,6^, Xuekui Yu^3,4,*^, Wenhu Duan ^2,3,*^, Zuoquan Xie^1,3*^

^1^State Key Laboratory of Drug Research, Shanghai Institute of Materia Medica, Chinese Academy of Sciences, Shanghai 201203, China.

^2^Small-Molecule Drug Research Center, Shanghai Institute of Materia Medica, Chinese Academy of Sciences, 555 Zu Chong Zhi Road, Shanghai 201203, China.

^3^University of Chinese Academy of Sciences, No. 19A Yuquan Road, Beijing 100049, China.

^4^Cryo-Electron Microscopy Research Center & The CAS Key Laboratory of Receptor Research, Shanghai Institute of Materia Medica, Chinese Academy of Sciences, Shanghai 201203, China

^5^Lingang Laboratory, Shanghai 200031, China

^6^Shandong Laboratory of Yantai Drug Discovery, Bohai Rim Advanced Research Institute for Drug Discovery, Yantai, Shandong 264117, China

**Chemical Synthesis**

*^a^*Reagents and conditions: (a) Pd(dppf)Cl_2_, Cs_2_CO_3_, THF/H_2_O, 70 ^o^C; (b) LiOH・H_2_O, THF/H_2_O.

**General procedure for the preparation of DW18320, DW10332, DW18333, DW18340, DW18342, DW18343, and DW18344.**

A mixture of methyl 4-oxobutanoate (**3a**–**3g**) (0.5 mmol, 1.0 eq), LiOH・H_2_O (2.5 mmol, 5.0 eq), THF (2.5 mL), and water (2.5 mL) was stirred at room temperature for 15 hours. The reaction mixture was treated with 1N hydrochloric acid, which was then extracted with ethyl acetate. The organic layer was separated and concentrated, and the residue was purified by silica gel chromatography (dichloromethane/methanol, v/v, 95:5 to 90:10) to give the desired product.

**4-(4-(Benzofuran-3-yl)furan-2-yl)-4-oxobutanoic acid (DW18320).**

Pale-yellow solid (61%), mp 153−155 °C. ^1^H NMR (400 MHz, DMSO-*d*_6_) *δ* 12.25 (br s, 1H), 8.66 (s, 1H), 8.49 (s, 1H), 8.03 (d, *J* = 6.0 Hz, 1H), 7.99 (s, 1H), 7.68 (d, *J* = 8.0 Hz, 1H), 7.36–7.44 (m, 2H), 3.14 (t, *J* = 6.7 Hz, 2H), 2.61 (t, *J* = 6.7 Hz, 2H); ^13^C NMR (150 MHz, DMSO-*d_6_*) *δ* 187.84, 174.14, 155.42, 152.46, 143.68, 143.65, 125.55, 125.37, 123.84, 121.31, 118.90, 116.93, 112.57, 112.19, 33.32, 27.98. HRMS (ESI) *m/z* calcd [M+H]^+^ for C_16_H_13_O_5_ 285.0763, found 285.0758.

**4-(4-(1*H*-indol-3-yl)thiophen-2-yl)-4-oxobutanoic acid (DW10332).**

White solid (69%), mp 174−175 °C. ^1^H NMR (400 MHz, DMSO-*d_6_*) *δ* 12.29 (br s, 1H), 11.42 (s, 1H), 8.39 (s, 1H), 8.12 (s, 1H), 7.97 (d, *J* = 8.0 Hz, 1H), 7.91 (d, *J* = 2.4 Hz, 1H), 7.46 (d, *J* = 8.0 Hz, 1H), 7.11–7.19 (m, 2H), 3.32 (t, *J* = 8.0 Hz, 2H), 2.62 (t, *J* = 8.0 Hz, 2H); ^13^C NMR (125 MHz, DMSO-*d_6_*) *δ* 191.78, 173.70, 142.96, 137.55, 136.58, 132.26, 125.29, 124.68, 124.23, 121.61, 119.74, 119.25, 111.89, 110.48, 33.48, 27.89. HRMS (ESI) *m/z* calcd [M+H]^+^ for C_16_H_14_NO_3_S 300.0694, found 300.0691.

**4-(4-(1*H*-indol-3-yl)furan-2-yl)-4-oxobutanoic acid (DW18333).**

Pale-brown solid (68%), mp 161−163 °C. ^1^H NMR (400 MHz, DMSO-*d*_6_) *δ* 12.23 (s, 1H), 11.38 (s, 1H), 8.46 (s, 1H), 7.94 (s, 1H), 7.88 (d, *J* = 8.0 Hz, 1H) , 7.83 (d, *J* = 2.4 Hz, 1H), 7.45 (d, *J* = 8.0 Hz, 1H), 7.09–7.19 (m, 2H), 3.13 (t, *J* = 6.4 Hz, 2H), 2.60 (t, *J* = 6.4 Hz, 2H); ^13^C NMR (150 MHz, DMSO-*d_6_*) *δ* 187.74, 174.20, 152.05, 141.69, 137.06, 125.09, 124.39, 123.08, 122.11, 120.03, 119.97, 117.30, 112.33, 106.23, 33.24, 28.08. HRMS (ESI) *m/z* calcd [M+H]^+^ for C_16_H_14_NO_4_ 284.0923, found 284.0921.

**4-(4-(6-Fluoro-1*H*-indol-3-yl)thiophen-2-yl)-4-oxobutanoic acid (DW18340).**

Yellow solid (75%), mp 176−178 °C. ^1^H NMR (400 MHz, DMSO-*d_6_*) *δ* 12.20 (br s, 1H), 11.49 (s, 1H), 8.39 (s, 1H), 8.14 (s, 1H), 7.94–8.00 (m, 1H), 7.92 (d, *J* = 4.0 Hz, 1H), 7.25 (dd, *J* = 4.0, 8.0 Hz, 1H), 7.01 (td, *J* = 4.0, 8.0 Hz, 1H), 3.30 (t, *J* = 6.4 Hz, 2H), 2.61 (t, *J* = 6.4 Hz, 2H); ^13^C NMR (125 MHz, DMSO-*d_6_*) *δ* 191.80, 173.70, 159.82 (d, *J* = 233.8 Hz), 143.06, 137.06, 136.58 (d, *J* = 12.3 Hz), 132.25, 125.57, 124.83, 121.56, 120.42 (d, *J* = 10.0 Hz), 110.66, 108.19 (d, *J* = 23.8 Hz), 97.94 (d, *J* = 25.0 Hz), 33.50, 27.92. HRMS (ESI) *m/z* calcd [M+H]^+^ for C_16_H_13_FNO_3_S 318.0600, found 318.0598.

**4-(4-(5-Fluoro-1*H*-indol-3-yl)thiophen-2-yl)-4-oxobutanoic acid (DW18342).**

Yellow solid (63%), mp 190−192 °C. ^1^H NMR (400 MHz, DMSO-*d_6_*) *δ* 12.18 (br s, 1H), 11.53 (s, 1H), 8.39 (d, *J* = 1.6 Hz, 1H), 8.15 (d, *J* = 1.6 Hz, 1H), 8.00 (d, *J* = 2.8 Hz, 1H), 7.76 (dd, *J* = 2.4, 10.2 Hz, 1H), 7.45 (q, *J* = 4.8, 8.8 Hz, 1H), 7.05 (td, *J* = 2.4, 10.2 Hz, 1H), 3.31 (t, *J* = 6.4 Hz, 2H), 2.60 (t, *J* = 6.4 Hz, 2H); ^13^C NMR (150 MHz, DMSO-*d_6_*) *δ* 192.34, 174.24, 158.84 (d, *J* = 230.9 Hz), 143.56, 137.52, 133.76, 132.65, 126.73, 125.94, 125.26 (d, *J* = 9.9 Hz), 113.42 (d, *J* = 9.9 Hz), 111.31 (d, *J* = 4.7 Hz), 110.36 (d, *J* = 26.1 Hz), 104.76 (d, *J* = 23.7 Hz), 34.00, 28.43. HRMS (ESI) *m/z* calcd [M+H]^+^ for C_16_H_13_FNO_3_S 318.0600, found 318.0598.

**4-(4-(7-Fluoro-1*H*-indol-3-yl)thiophen-2-yl)-4-oxobutanoic acid (DW18343).**

Pale-yellow solid (67%), mp 193−194 °C. ^1^H NMR (600 MHz, DMSO-*d_6_*) *δ* 12.21 (s, 1H), 11.92 (s, 1H), 8.43 (d, *J* = 1.2 Hz, 1H), 8.15 (d, *J* = 1.2 Hz, 1H), 8.00 (d, *J* = 2.4 Hz, 1H), 7.80 (d, *J* = 7.8 Hz, 1H), 7.09–7.12 (m, 1H), 7.01–7.04 (m, 1H), 3.32 (t, *J* = 6.6 Hz, 2H), 2.63 (t, *J* = 6.6 Hz, 2H); ^13^C NMR (150 MHz, DMSO-*d_6_*) *δ* 192.27, 174.19, 150.63 (d, *J* = 241.5 Hz), 143.61, 137.34, 132.82, 129.07 (d, *J* = 6.0 Hz), 126.44, 125.83, 125.01 (d, *J* = 13.5 Hz), 120.62 (d, *J* = 6.0 Hz), 116.04 (d, *J* = 3.0 Hz), 112.08 (d, *J* = 3.0 Hz), 107.04 (d, *J* = 16.5 Hz), 33.98, 28.36. HRMS (EI) *m/z* calcd M^+^ for C_16_H_12_FNO_3_S 317.0522, found 317.0515.

**4-(4-(4-fluoro-1*H*-indol-3-yl)thiophen-2-yl)-4-oxobutanoic acid (DW18344).**

Yellow solid (63%), mp 168−170 °C. ^1^H NMR (400 MHz, DMSO-*d_6_*) *δ* 12.14 (br s, 1H), 11.77 (s, 1H), 8.30 (t, *J* = 1.2 Hz, 1H), 7.96 (t, *J* = 1.2 Hz, 1H), 7.85 (d, *J* = 2.8 Hz, 1H), 7.30 (d, *J* = 8.0 Hz, 1H), 7.11–7.16 (m, 1H), 6.89 (q, *J* = 7.6, 12.0 Hz, 1H), 3.26 (t, *J* = 6.6 Hz, 2H), 2.61 (t, *J* = 6.6 Hz, 2H); ^13^C NMR (150 MHz, DMSO-*d_6_*) *δ* 192.21, 174.26, 157.14 (d, *J* = 242.7 Hz), 143.20, 140.01 (d, *J* = 11.4 Hz), 137.09, 133.97 (d, *J* = 4.2 Hz), 128.05 (d, *J* = 10.5 Hz), 125.71, 122.73 (d, *J* = 8.3 Hz), 113.55 (d, *J* = 19.2 Hz), 109.51, 109.04 (d, *J* = 3.3 Hz), 105.27 (d, *J* = 20.9 Hz), 33.99, 28.46. HRMS (ESI) *m/z* calcd [M+H]^+^ for C_16_H_13_FNO_3_S 318.0600, found 318.0595.

**General procedure for the preparation of intermediates 3a–3g.**

A mixture of aryl bromine (**1a**–**1b**) (1.0 mmol, 1.0 eq), boric acid ester (**2a**–**2f**) (1.2 mmol, 1.2 eq), cesium carbonate (2.5 mmol, 2.5 eq), Pd(dppf)Cl_2_ (0.05 mmol, 0.05 eq), THF (3 mL), and water (3 mL) was purged with argon for 10 minutes and then heated at 70 °C under argon for 15 hours. After cooling to room temperature, water was added to the reaction mixture and it was then extracted with dichloromethane. The organic layer was concentrated in vacuum, and the residue was purified by silica gel chromatography (petroleum ether/ethyl acetate, v/v, 99:1 to 90:10) to give the desired product (**3a**–**3g**).

**Methyl 4-(4-(benzofuran-3-yl)furan-2-yl)-4-oxobutanoate (3a).**

Pale-brown solid (65%), mp 101−103 °C. ^1^H NMR (400 MHz, CDCl_3_) *δ* 7.94 (s, 1H), 7.82 (s, 1H), 7.71 (d, *J* = 8.0 Hz, 1H), 7.57 (d, *J* = 8.0 Hz, 1H), 7.49 (s, 1H), 7.32–7.40 (m, 2H), 3.72 (s, 3H), 3.27 (t, *J* = 6.6 Hz, 2H), 2.81 (t, *J* = 6.6 Hz, 2H). LRMS (ESI) *m/z* calcd [M+H]^+^ for C_17_H_15_O_5_ 299.1, found 299.1.

**Tert-butyl 3-(5-(4-methoxy-4-oxobutanoyl)thiophen-3-yl)-1H-indole-1-carboxylate (3b).**

Yellow oil (76%). ^1^H NMR (400 MHz, CDCl_3_) *δ* 8.24 (d, *J* = 8.0 Hz, 1H), 8.03 (d, *J* = 1.6 Hz, 1H), 7.77–7.80 (m, 3H), 7.31–7.42 (m, 2H), 3.73 (s, 3H), 3.35 (t, *J* = 8.0 Hz, 2H), 2.83 (t, *J* = 8.0 Hz, 2H), 1.71 (s, 9H). LRMS (ESI) *m/z* calcd [M+H]^+^ for C_22_H_24_NO_5_S 414.1, found 414.1.

**Tert-butyl 3-(5-(4-methoxy-4-oxobutanoyl)furan-3-yl)-1H-indole-1-carboxylate (3c).**

White solid (71%), mp 90−92 °C. ^1^H NMR (400 MHz, CDCl_3_) *δ* 8.23 (d, *J* = 12.0 Hz, 1H), 7.94 (s, 1H), 7.73 (s, 1H), 7.69 (d, *J* = 8.0 Hz, 1H), 7.52 (s, 1H), 7.31–7.41 (m, 2H), 3.72 (s, 3H), 3.25 (t, *J* = 6.8 Hz, 2H), 2.79 (t, *J* = 6.8 Hz, 2H), 1.70 (s, 9H). LRMS (ESI) *m/z* calcd [M+H]^+^ for C_22_H_24_NO_6_ 398.2, found 398.1.

**Tert-butyl 6-fluoro-3-(5-(4-methoxy-4-oxobutanoyl)thiophen-3-yl)-1H-indole-1-carboxylate (3d).**

Pale-brown solid (71%), mp 96−98 °C. ^1^H NMR (400 MHz, CDCl_3_) *δ* 7.99 (s, 1H), 7.93–7.96 (d, *J* = 12.0 Hz, 1H), 7.65–7.77 (m, 3H), 7.10 (td, *J* = 4.0, 8.0 Hz, 1H), 3.72 (s, 3H), 3.32 (t, *J* = 6.6 Hz, 2H), 2.81 (t, *J* = 6.6 Hz, 2H), 1.70 (s, 9H). LRMS (ESI) *m/z* calcd [M+H]^+^ for C_22_H_23_FNO_5_S 432.1, found 432.0.

**Tert-butyl 5-fluoro-3-(5-(4-methoxy-4-oxobutanoyl)thiophen-3-yl)-1H-indole-1-carboxylate (3e).**

Yellow solid (63%), mp 106−108 °C. ^1^H NMR (400 MHz, CDCl_3_) *δ* 8.18–8.19 (m, 1H), 7.98 (d, *J* = 1.6 Hz, 1H), 7.75–7.79 (m, 2H), 7.44 (dd, *J* = 2.4, 9.2 Hz, 1H), 7.14 (td, *J* = 2.4, 9.2 Hz, 1H), 3.73 (s, 3H), 3.33 (t, *J* = 6.7 Hz, 2H), 2.81 (t, *J* = 6.7 Hz, 2H), 1.70 (s, 9H). LRMS (ESI) *m/z* calcd [M+H]^+^ for C_22_H_23_FNO_5_S 432.1, found 432.0.

**Tert-butyl 7-fluoro-3-(5-(4-methoxy-4-oxobutanoyl)thiophen-3-yl)-1H-indole-1-carboxylate (3f).**

Yellow oil (63%). ^1^H NMR (400 MHz, CDCl_3_) *δ* 7.99 (d, *J* = 1.6 Hz, 1H), 7.79 (s, 1H), 7.77 (d, *J* = 1.6 Hz, 1H), 7.55 (dd, *J* = 0.8, 8.0 Hz, 1H), 7.24–7.29 (m, 1H), 7.14 (q, *J* = 8.0, 12.8 Hz, 1H), 3.73 (s, 3H), 3.33 (t, *J* = 6.8 Hz, 2H), 2.81 (t, *J* = 6.8 Hz, 2H), 1.68 (s, 9H). LRMS (ESI) *m/z* calcd [M+H]^+^ for C_22_H_23_FNO_5_S 432.1, found 432.2.

**Tert-butyl 4-fluoro-3-(5-(4-methoxy-4-oxobutanoyl)thiophen-3-yl)-1H-indole-1-carboxylate (3g).**

Yellow oil (63%). ^1^H NMR (400 MHz, CDCl_3_) *δ* 8.06 (d, *J* = 8.4 Hz, 1H), 8.01 (t, *J* = 2.0 Hz, 1H), 7.82 (t, *J* = 2.0 Hz, 1H), 7.68 (s, 1H), 7.28–7.33 (m, 1H), 7.01 (qd, *J* = 0.8, 8.0, 11.2 Hz, 1H), 3.72 (s, 3H), 3.32 (t, *J* = 6.8 Hz, 2H), 2.80 (t, *J* = 6.8 Hz, 2H), 1.70 (s, 9H). LRMS (ESI) *m/z* calcd [M+H]^+^ for C_22_H_23_FNO_5_S 432.1, found 432.1.

**Structural Identification Data of Compound DW18320**


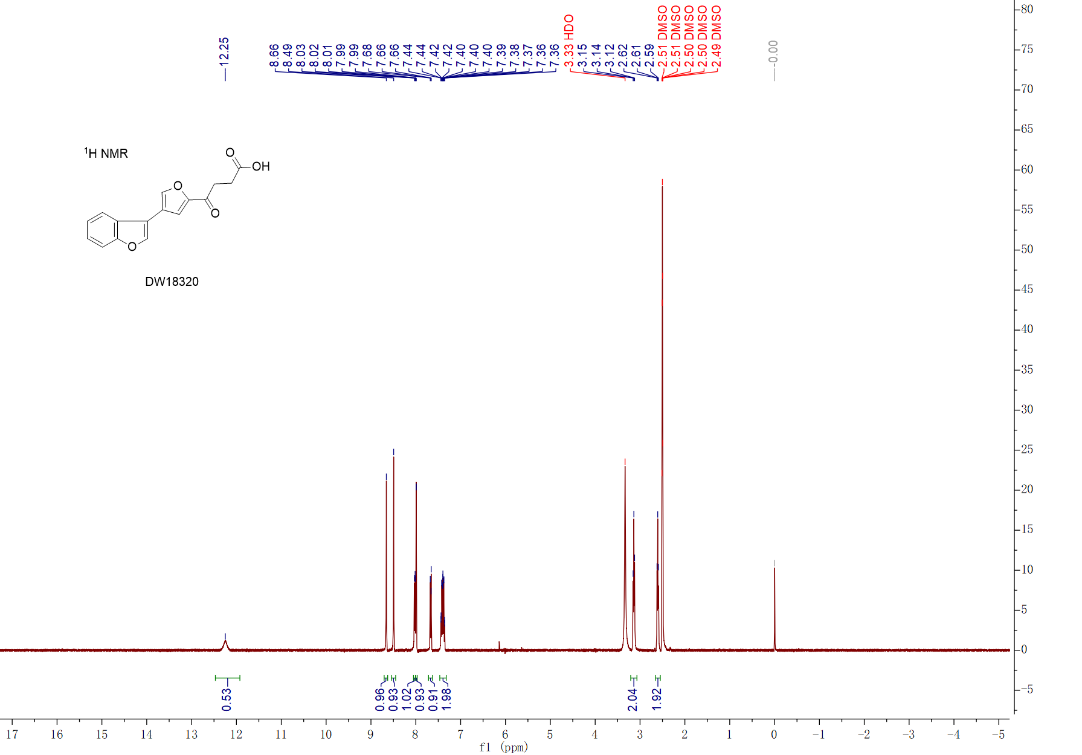


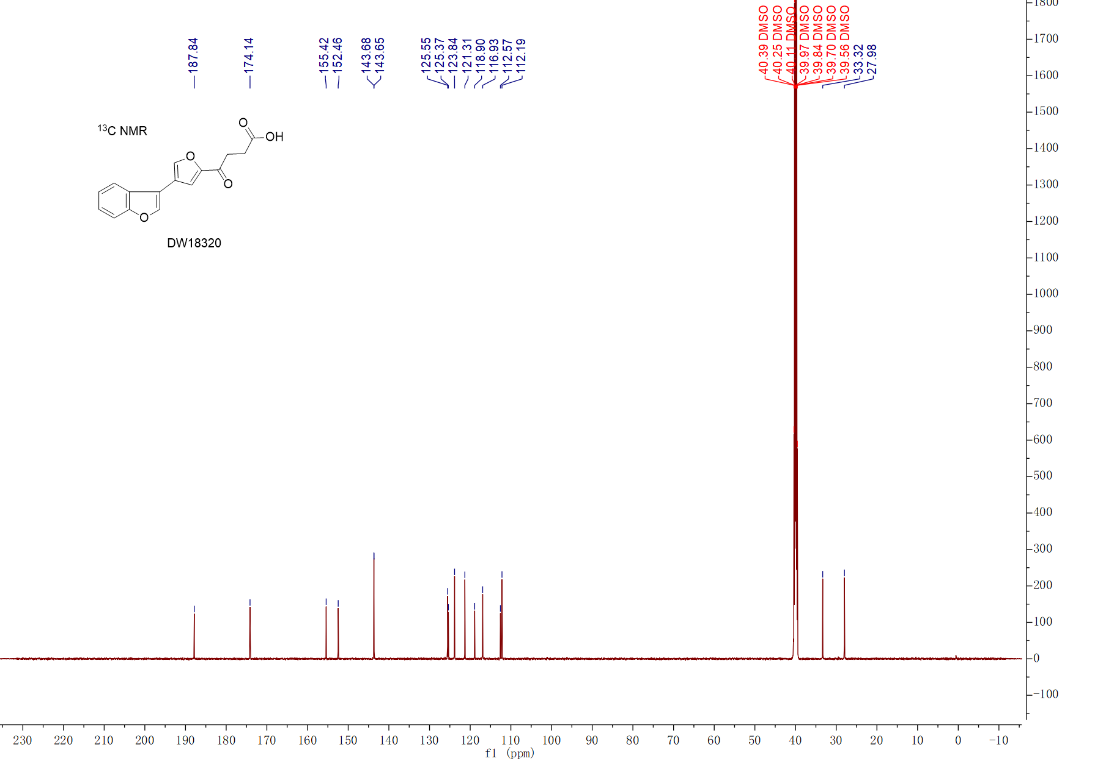


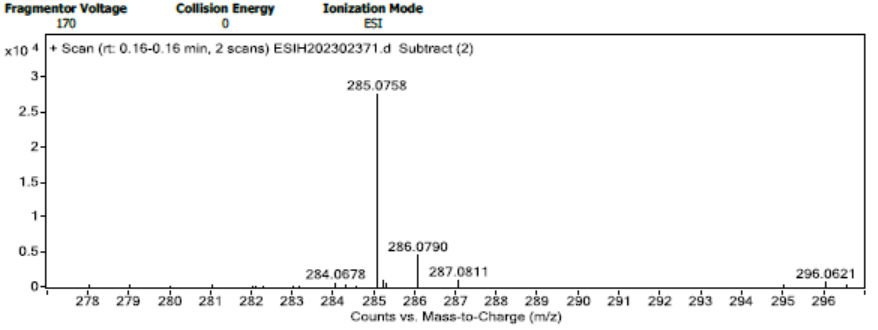


**Structural Identification Data of Compound DW10332**


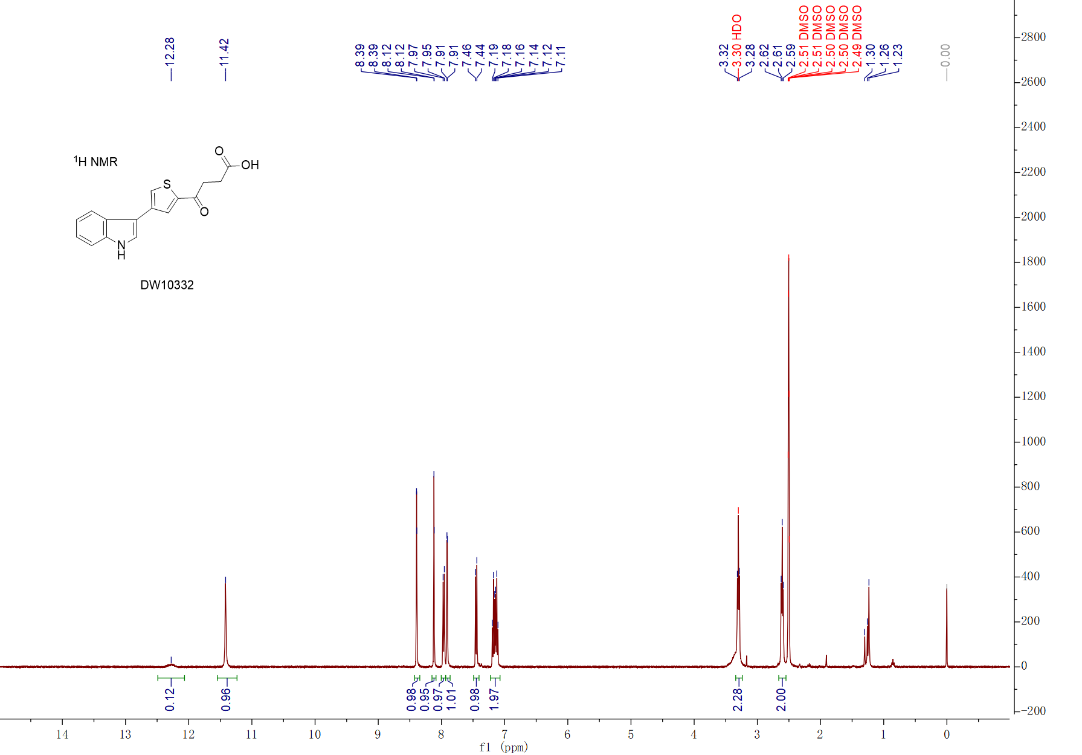


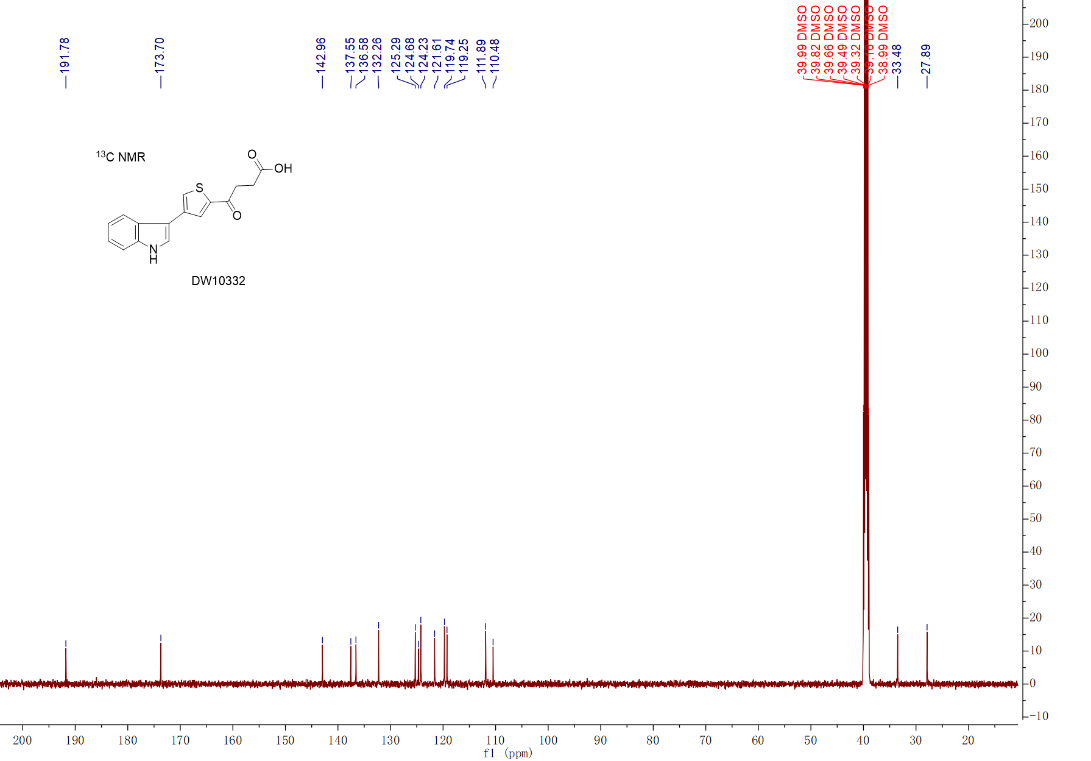


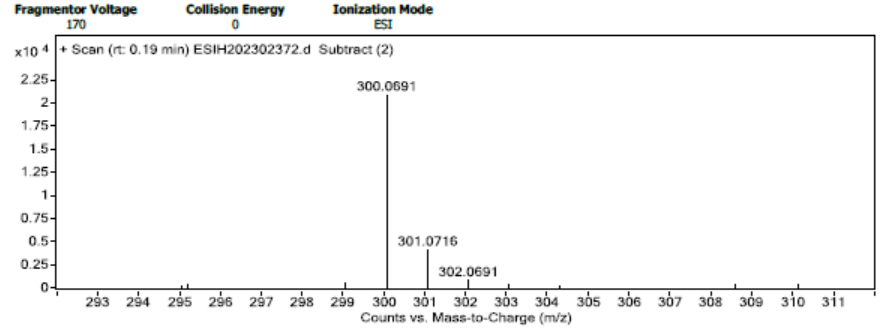


**Structural Identification Data of Compound DW18333**


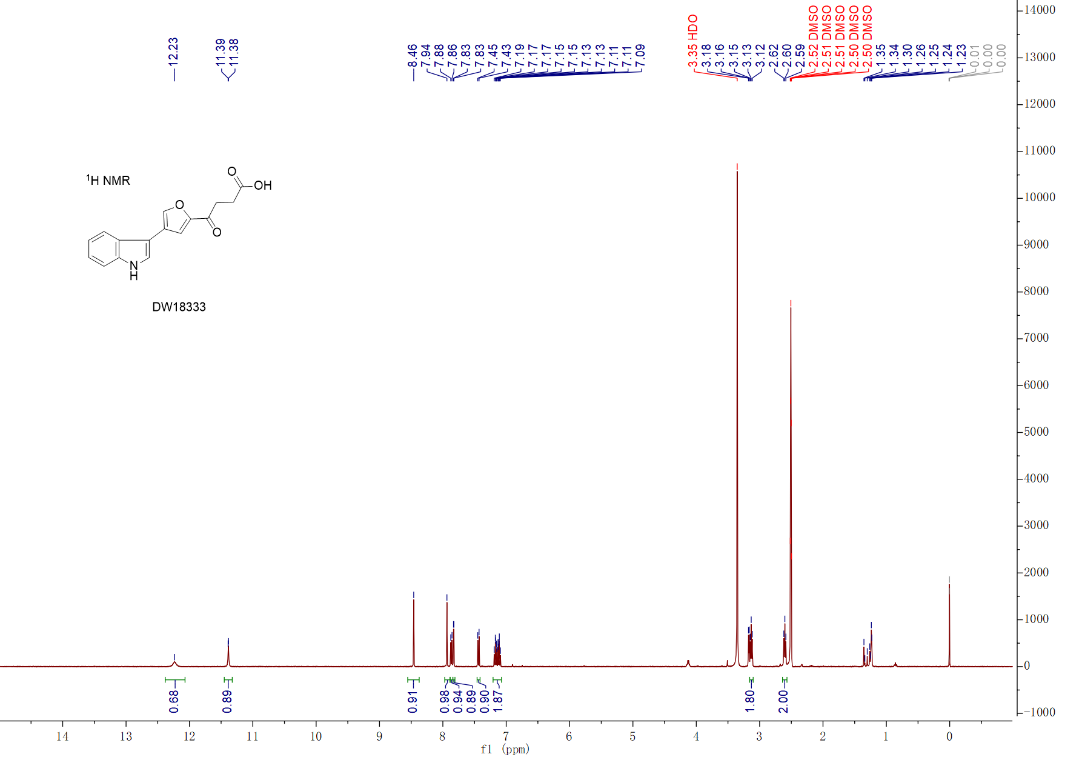


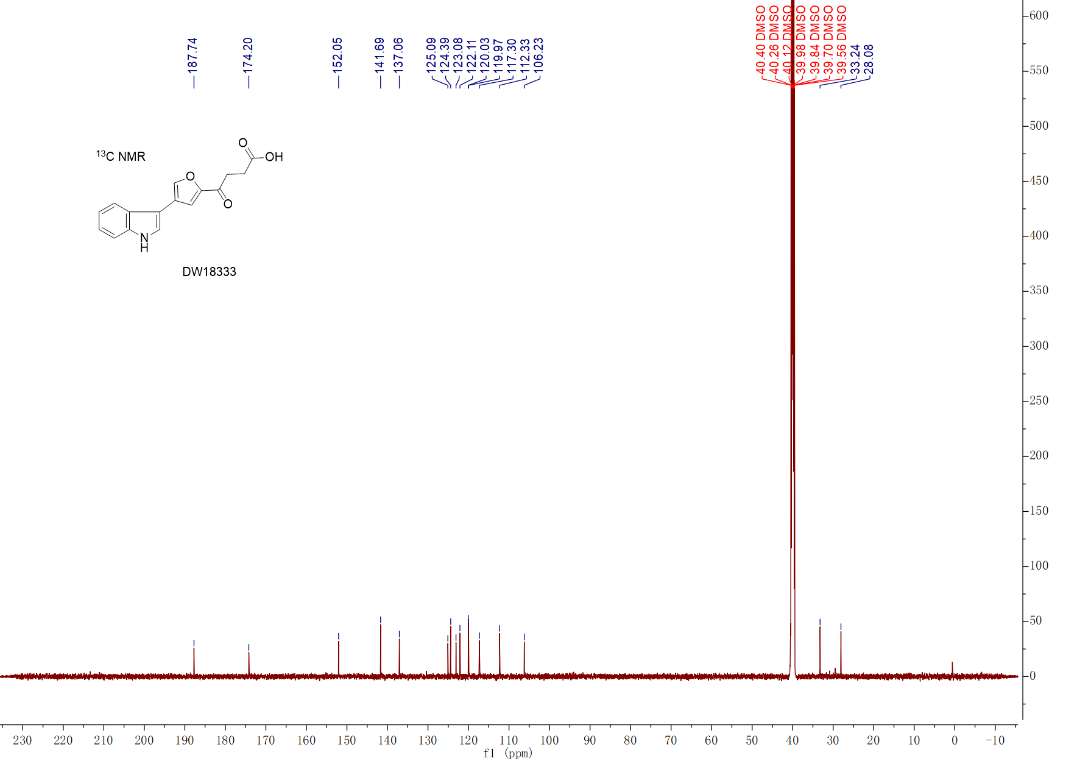


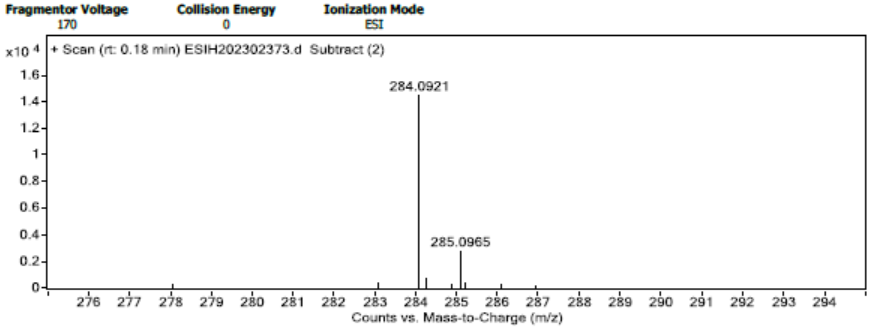


**Structural Identification Data of Compound DW18340**


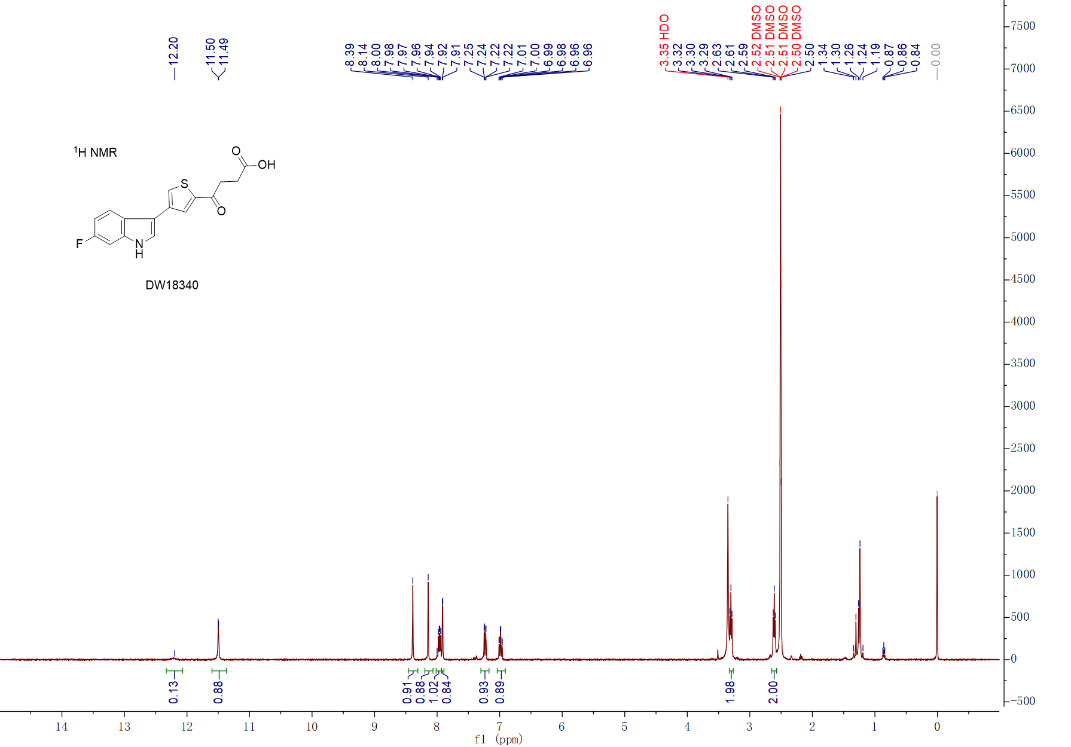


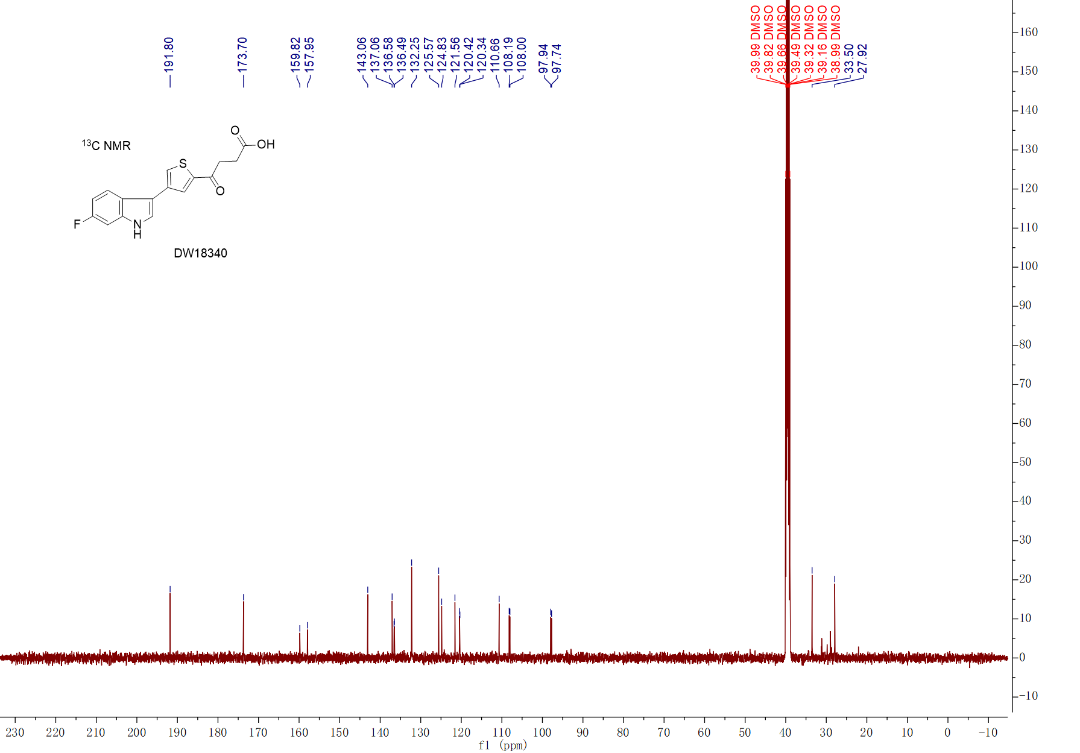


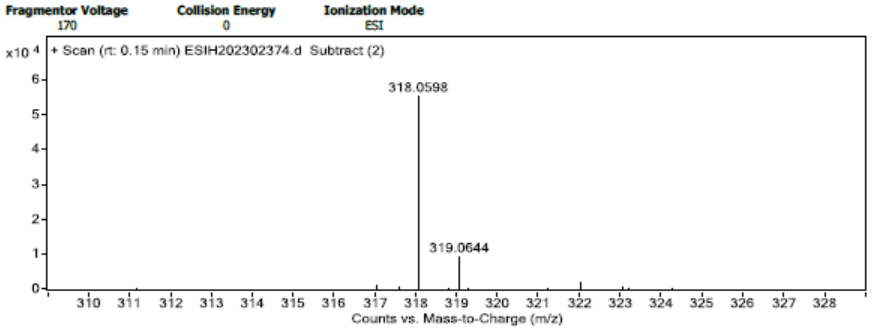


**Structural Identification Data of Compound DW18342**


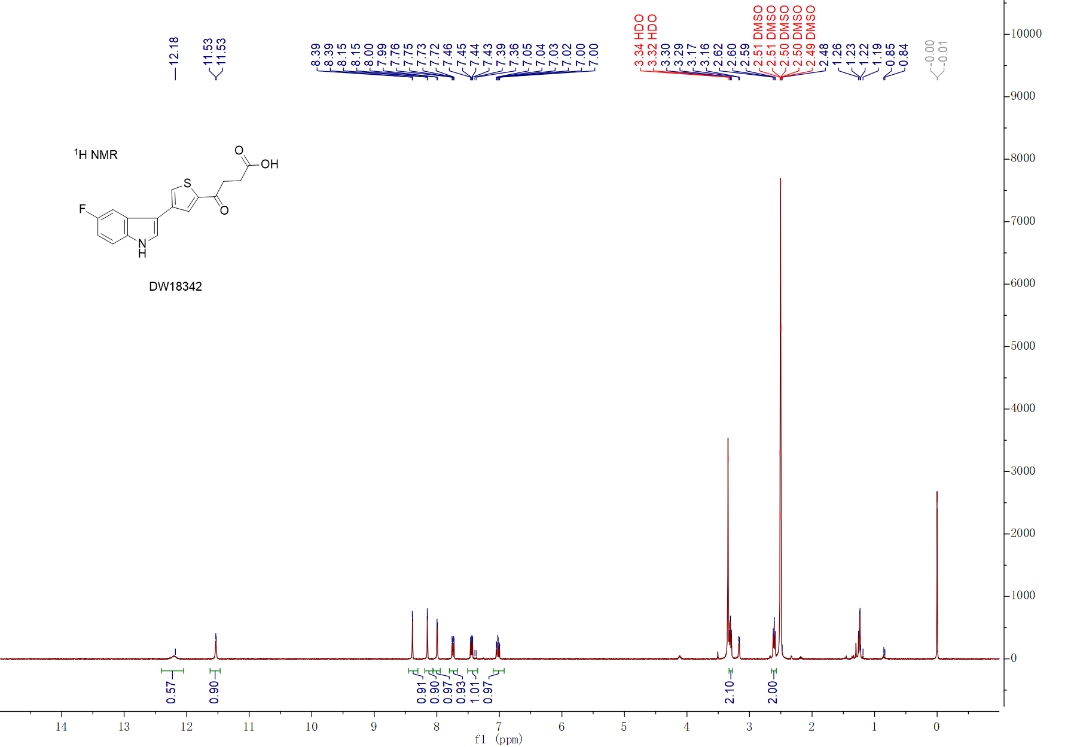


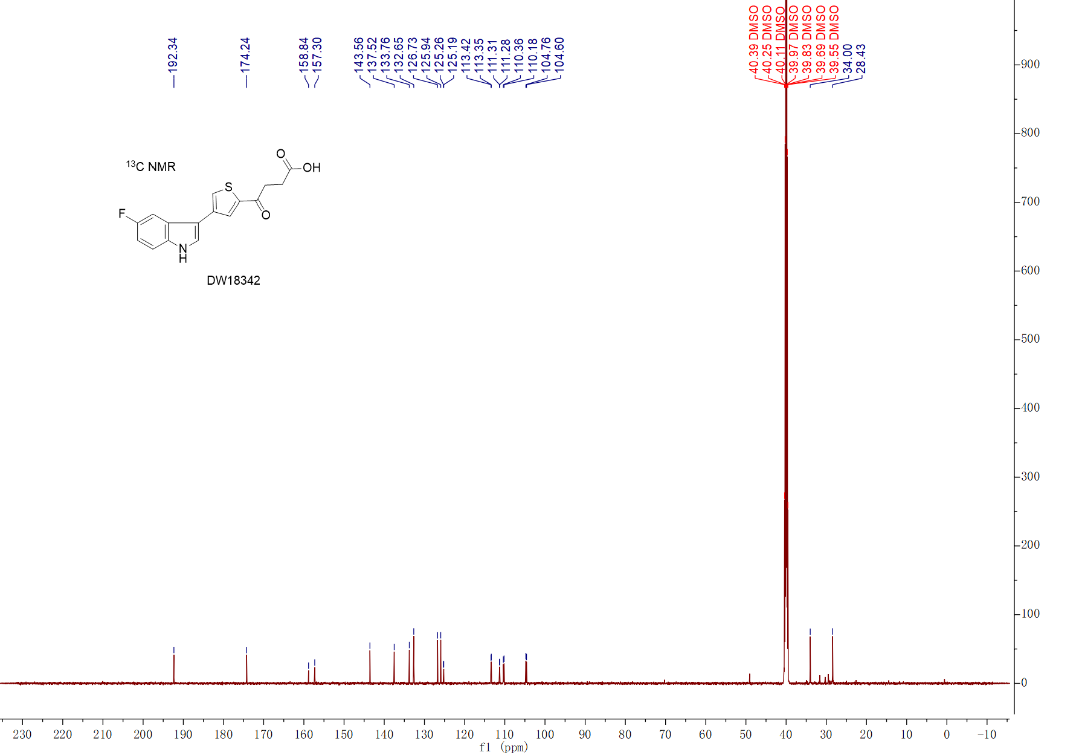


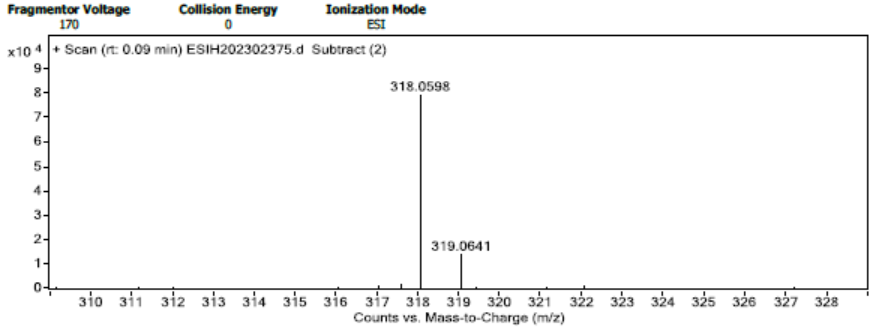


**Structural Identification Data of Compound DW18343**


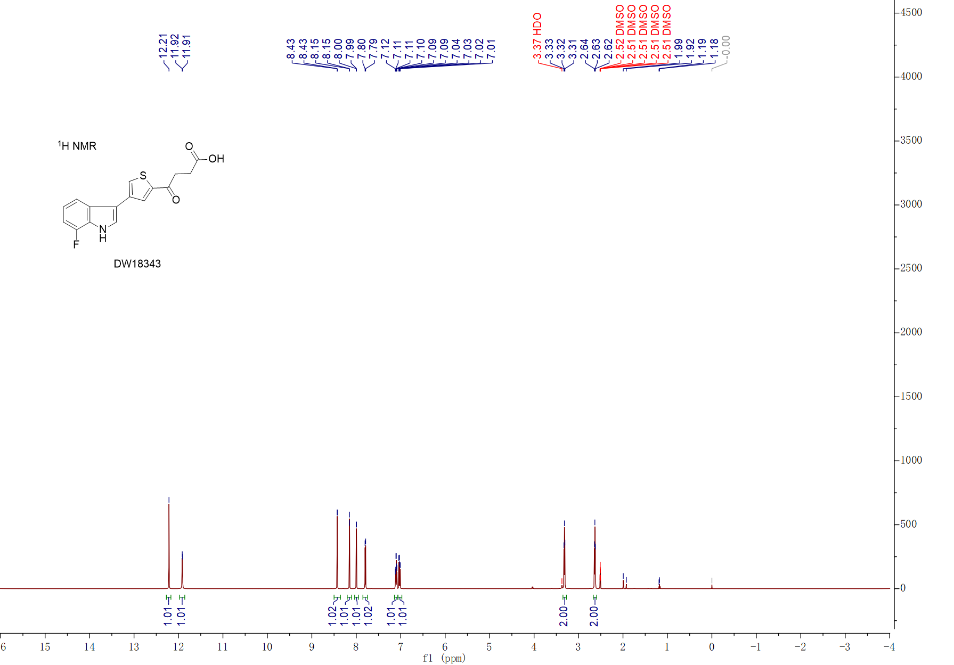


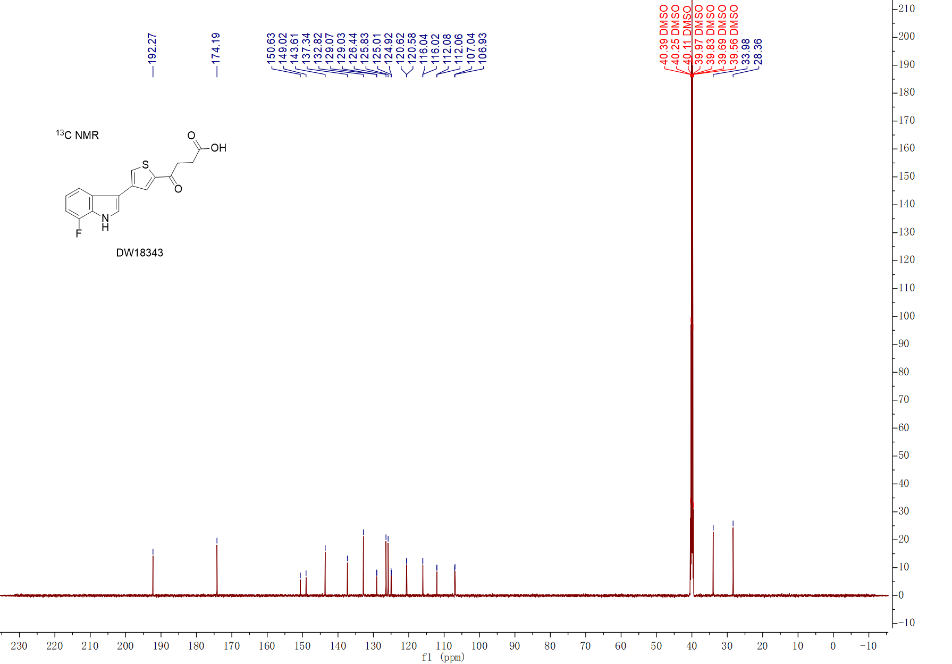


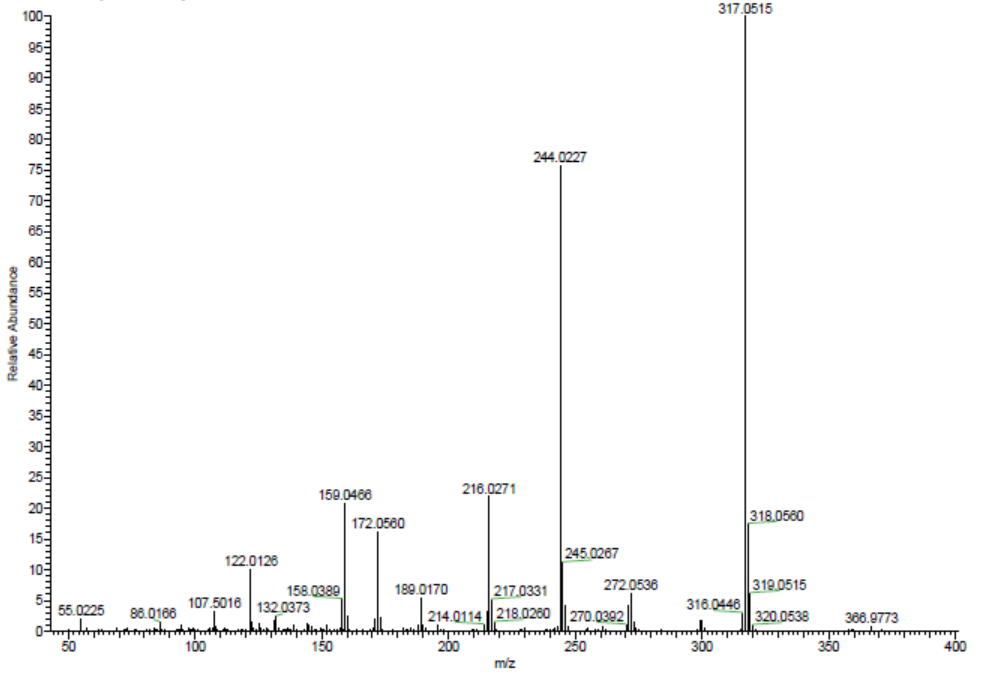


**Structural Identification Data of Compound DW18344**


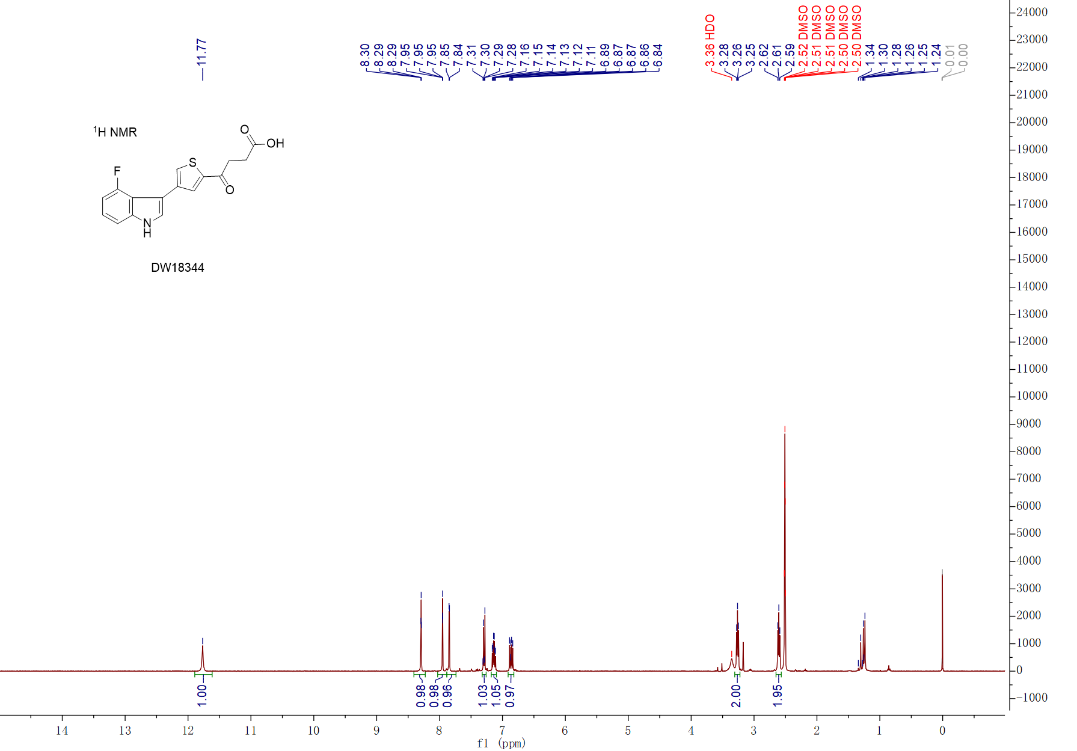


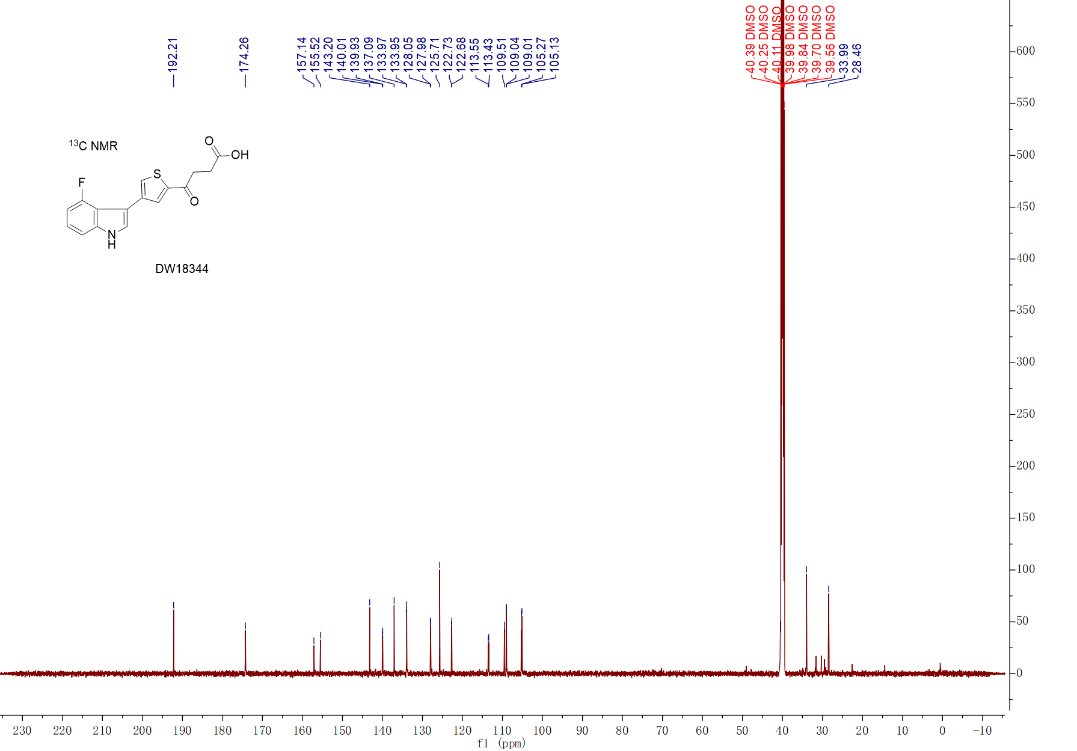


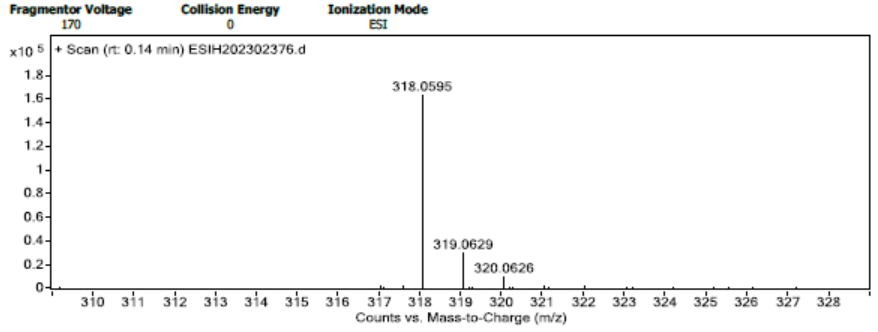


**Supplementary Methods**

**Cell viability assay**

THP1-Dual cells were seeded into 96-well plates at a density of 5×10³ cells per 180 µL. Then, 20 µL of DW18343 or S100 at specified concentrations was added to each well. The cells were incubated for 72 hours. Then, 20 µL of Cell-Counting Kit (AC11L054, Life-iLab Co., Ltd.) was added to each well, and absorbance at 450 nm was measured using a SpectraMAX Plus 384 instrument (Molecular Devices, Sunnyvale, CA). The experiments were conducted in triplicate.

**Analysis of peripheral blood immune cell subsets**

Peripheral immune cells were collected from the blood of C57/BL6 mice (n=7). Red blood cells were removed using a lysis buffer (Cat: MA0207, Meilun Bio, China), leaving white blood cells for subsequent experiments. The white blood cells were cultured with RPMI-1640 medium containing 10%FBS and treated with vehicle (0.1% DMSO) or DW18343 (10 μM) for 72 hours. Cells were transferred into Corning R 96-well Clear V-Bottom TC treated Microplates (Cat: 3894, Corning) to reduce their loss during multiple centrifugations. Next, the cells were re-suspended in FACS buffer and exposed to surface antibodies for 20 minutes in the dark. Zombie NIR Fixable Viability Kit (Cat: 423105, Biolegend) was used for a 15-minute dark incubation to identify live cells. Surface antibodies included CD45 (Cat: 157214, Biolegend), CD3 (Cat: 100233, Biolegend), CD8 (Cat: 367-0081-82, ThermoFisher), CD11b (Cat: 363-0112-82, ThermoFisher), Ly6C (Cat: 560525, BD), Ly6G (Cat: 562737, BD), F4/80 (Cat: 743280, BD), CD86 (Cat: 159204, Biolegend) and CD206 (Cat: 141708, Biolegend). For intracellular protein staining, the cells were fixed, permeabilized, and stained with antibodies against intracellular molecules, including granzyme B (GZMB) (Cat: 396414, Biolegend) and interferon-γ (IFNG) (Cat: 163508, Biolegend). Following staining, the samples were fixed with 1% paraformaldehyde in the dark for 15 minutes. After washing, each sample was re-suspended in FACS buffer and analyzed using BD LSRFortessa. The raw data collected were processed using Flowjo software.

**Supplementary Tables**

**Table S1. Data collection and refinement statistics.**

|  | **STING LBD with DW18343** |
| --- | --- |
| **Data collection** |  |
| Space group | *P41212* |
| **Cell dimensions** |  |
| a, b, c (Å) | 110.04, 110.04, 35.76 |
| α, β, γ (°) | 90.0, 90.0, 90.0 |
| Resolution (Å) | 34.8-1.81 (1.86-1.81)* |
| *R*_merge_ (%) | 8.3 (46.8) |
| Mean *I*/σ*I* | 11.5 (1.2) |
| Completeness (%) | 99.87 (99.9) |
| Redundancy | 4.1 (5.0) |
| CC1/2 | (0.345) |
| **Refinement** |  |
| Resolution (Å) | 34.798-1.810 |
| No. reflections | 20598 |
| *R*_work_/*R*_free_ (%) | 19.66/23.30 |
| **No. atoms** |  |
| Protein | 1413 |
| Water | 118 |
| Ligand | 22 |
| **B factors** |  |
| Protein | 31.7 |
| Water | 39.2 |
| Ligand | 16.3 |
| **R.m.s deviations** |  |
| Bond lengths (Å) | 0.0091 |
| Bond angles (°) | 1.12 |

^*^Values in parentheses indicate the highest resolution shell

**Table S2. Pharmacokinetics parameters of DW18343 in mice.**

| **Compound** | **Dose** | **Cmax** | **Tmax** | **T1/2** | **AUC0→∞** | **CL** | **Vss** | **F** |
| --- | --- | --- | --- | --- | --- | --- | --- | --- |
|  | **(mg/kg)** | **(μg/mL)** | **(h)** | **(h)** | **(****μg·h/mL)** | **(mL/min/kg)** | **(L/kg)** | **(%)** |
| **DW18343** | 5 (IV) | 7.69±2.27 | 0.05 | 0.86±0.71 | 1.44±0.24 | 59.0±11.1 | 0.78±0.22 |  |
|  | 5 (SC) | 4.82±2.27 | 0.12 | 0.53±0.25 | 1.45±0.41 | - | - | 100 |

**Supplementary Figures**


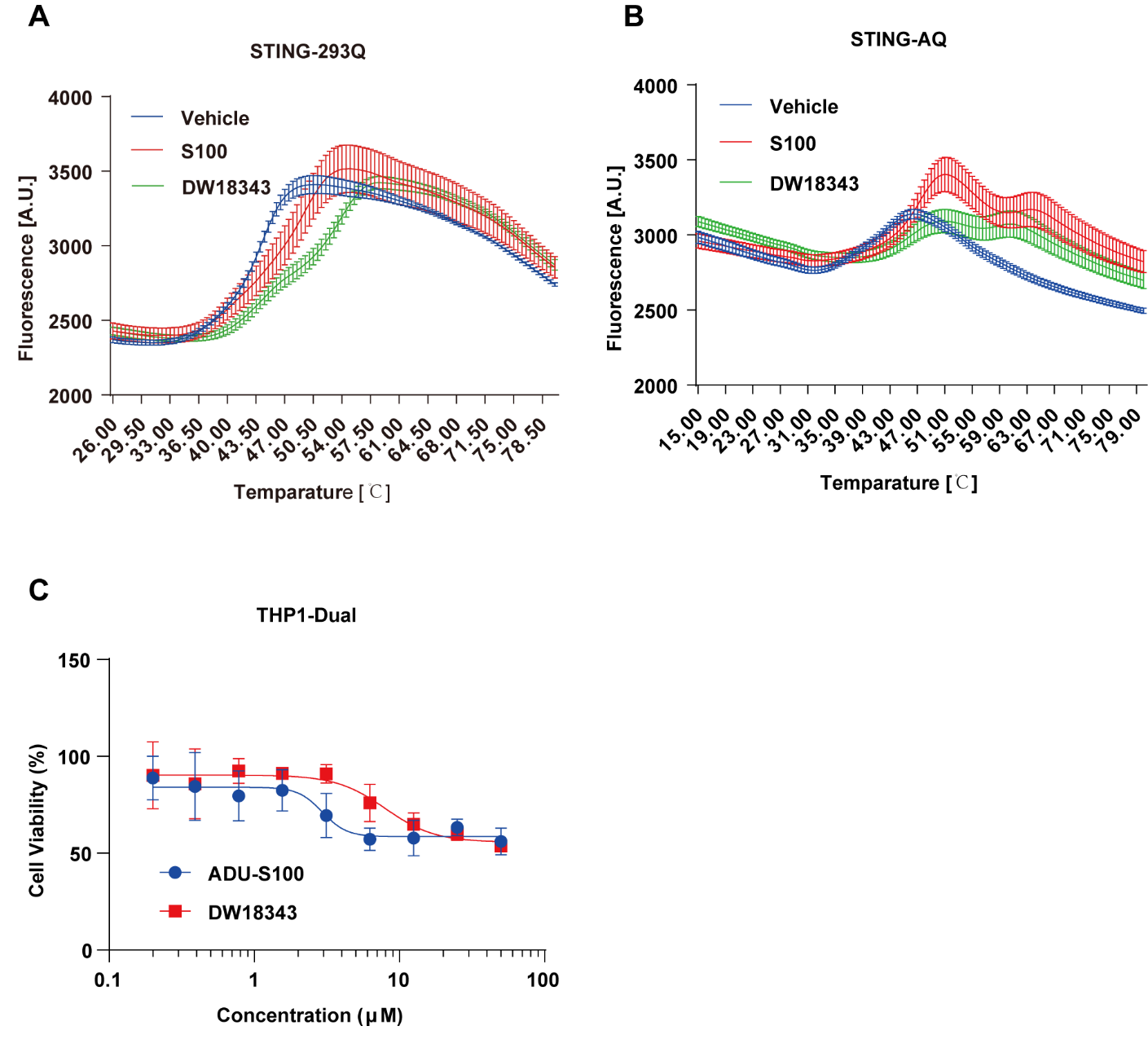


**Figure S1. Binding of compound on STING isoforms. A, B** Evaluation of the thermal stability of compound binding to human STING isoforms STING-293Q(**A**) and STING-AQ (**B**) using DSF, depicted as the Mean ± SEM of fluorescence value. **C** THP1-Dual cells were treated with serial doses of DW18343 or S100 for 72 hours. Cell viability was subsequently assessed using the CCK8 assay, and results were expressed as a percentage relative to the control group, presented as the mean ± SEM, n=3.


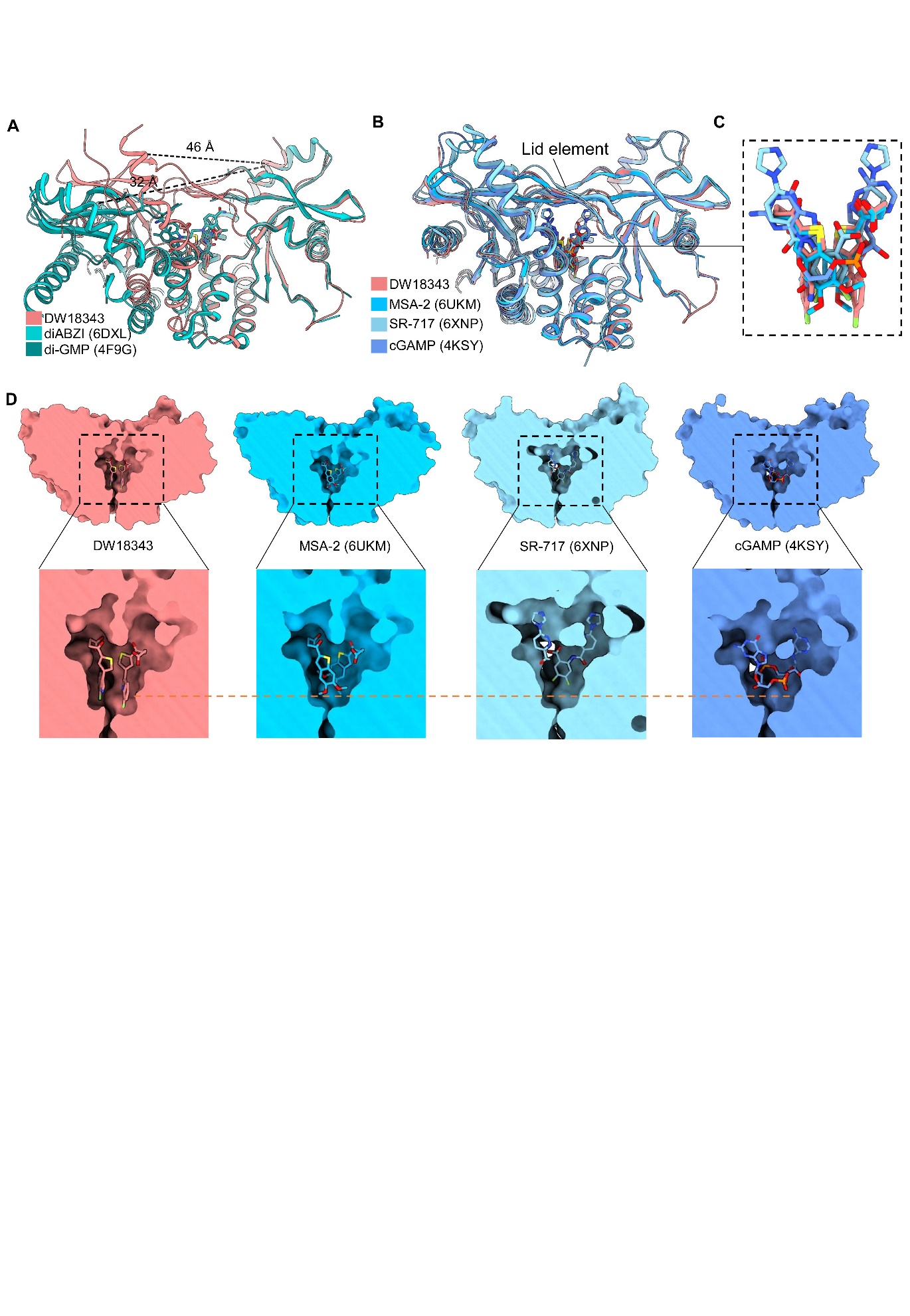


**Figure S2. Structural comparison of DW18343-bound LBD with another agonist-bound LBD. A** Superposition of the structure of DW18343-bound LBD with diABZI-bound (PDB ID 6DXL) and di-GMP-bound (PDB ID 4F9G) LBD. The distances between two LBDs are labeled. The distances in the structures of diABZI and di-GMP-bound LBD is similar, and only one in the structure of diABZI-bound LBD is showed for clarify. **B** Superposition of the structures of DW18343-bound LBD with MSA-2 (PDB ID 6UKM), SR-717 (PDB ID 6XNP), and cGAMP (PDB ID 4KSY)-bound LBD. **C** Zoomed view showing the binding poses of those agonists in (**B**). **D** Cross-section view showing the spatial position of agonists in the pocket formed by two LBDs. The agonists including MSA-2, SR-717, and cGAMP are located above the yellow dashed line.

**
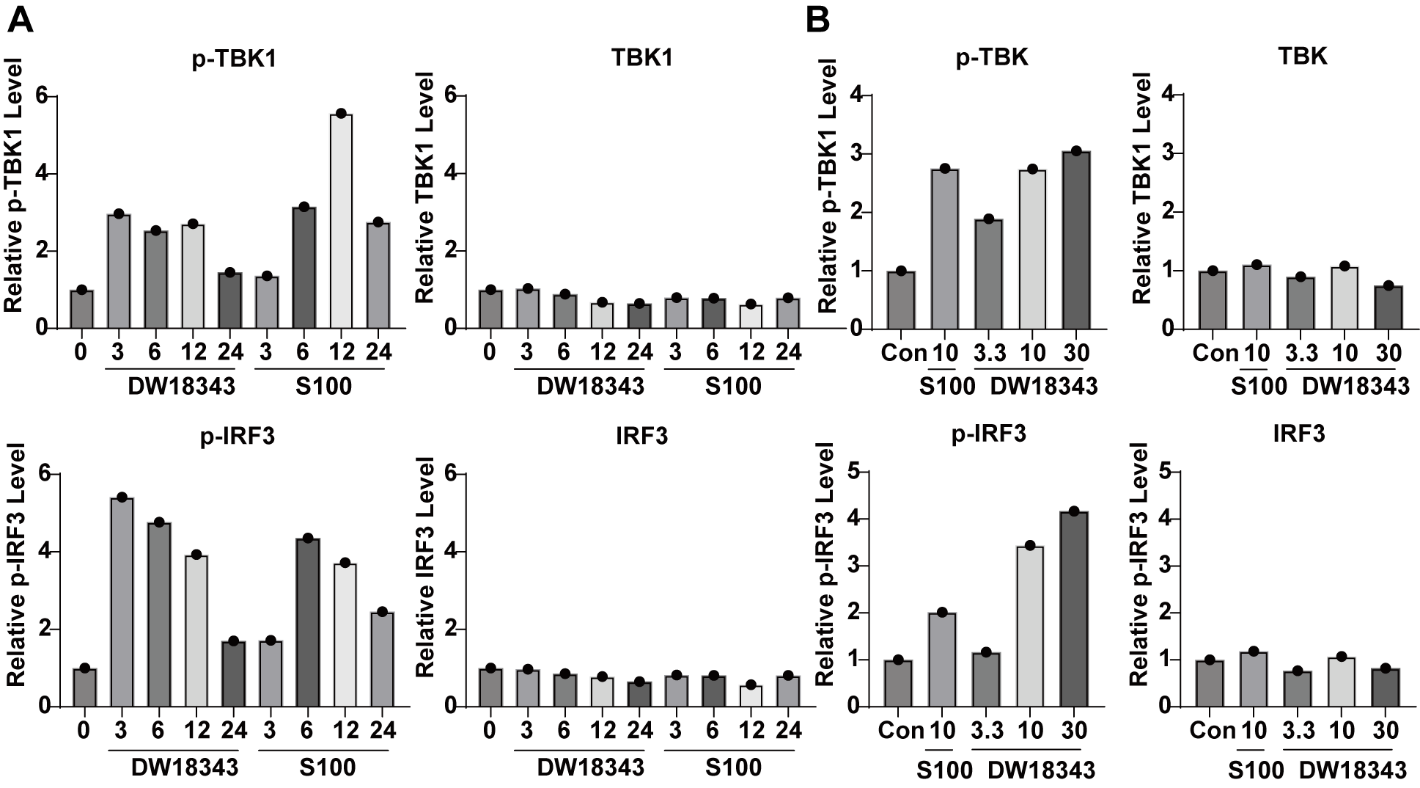
**

**Figure S3.** The grayscale values of the Western Blot bands in Figure 3A (A) and Figure 3B (B), respectively.

**
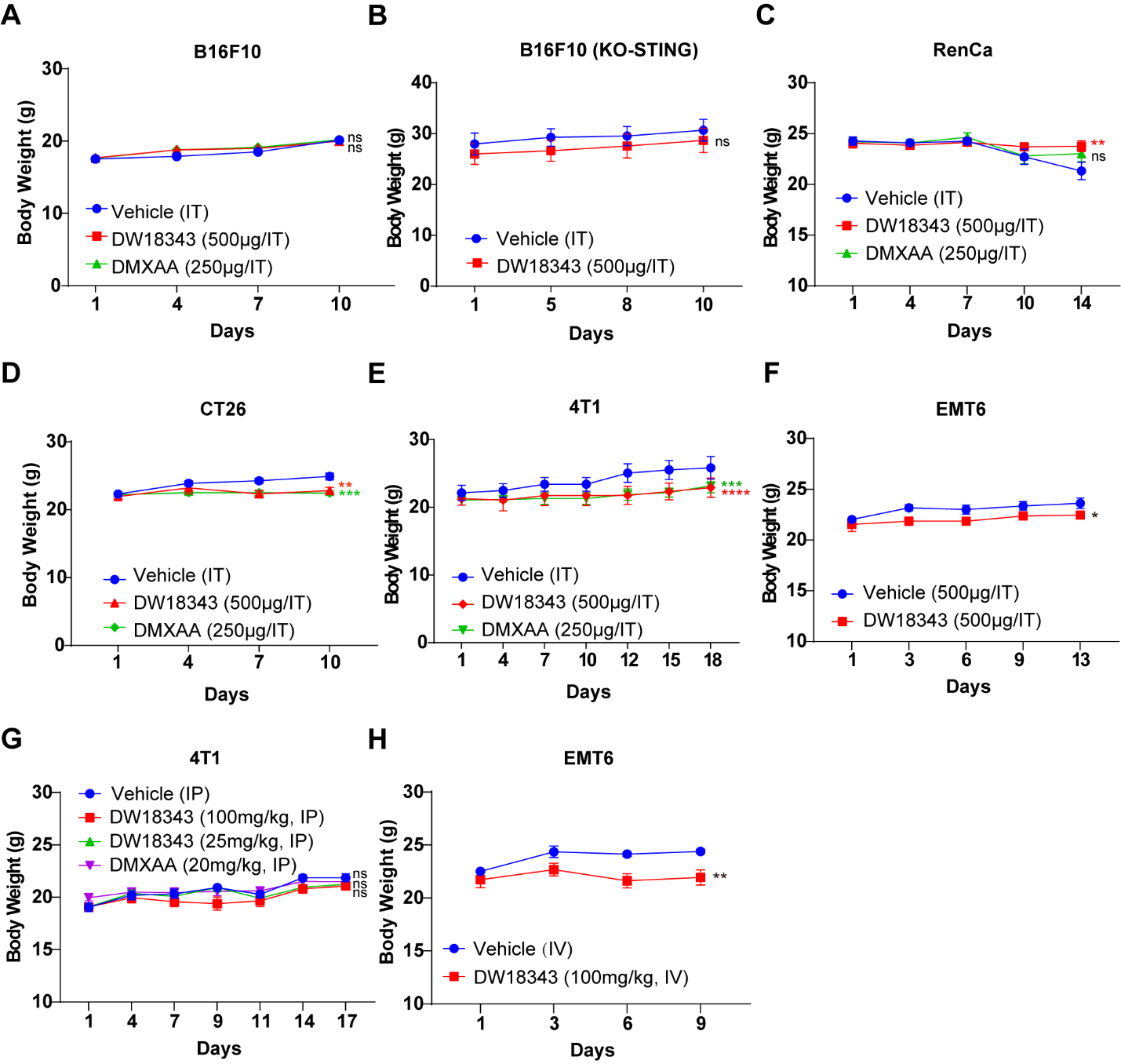
**

**Figure S4. The body weight changes of mice during compound treatment. A, B** Body weight changes of mice during DW18343 treatment in B16F10 tumors of C57/BL6 and C57/BL6 KO-STING mice, as referred to in Figure 4A and B. **C-F**. Body weight changes of mice during DW18343 treatment in RenCa, CT26, 4T1, and EMT6 tumors of Balb/c mice, as referred to in Figure 4C-F. **G** Body weight changes of mice during DW18343 treatment in 4T1 tumors of Balb/c mice, as referred to in Figure 4G. **H** Body weight changes of mice during DW18343 treatment in EMT6 tumors of Balb/c mice, as referred to in Figure 4H. A two-way analysis of variance (ANOVA) was performed to determine differences between treated groups and vehicle control, ns, no significant difference, ^**^*P* < 0.01, ^***^*P* < 0.001, and ^****^*P*< 0.0001.


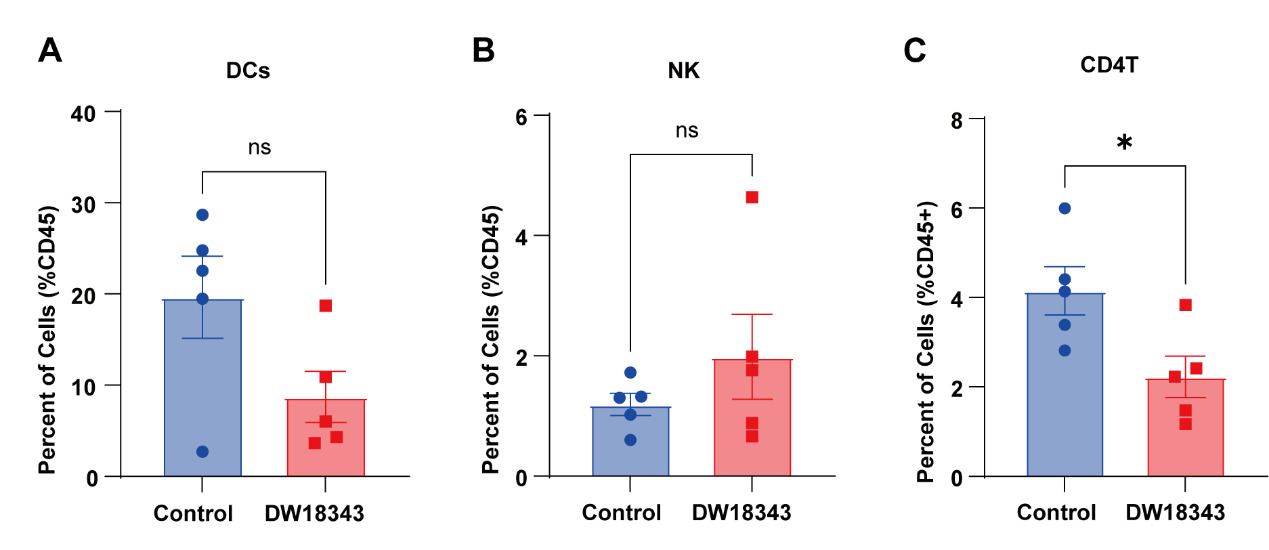


**Figure S5.** **Alterations in immune cell subpopulations in tumor tissues during DW18343 treatment. A-C** The RenCa tumor-bearing mice were intratumorally treated with 40% PEG400 or DW18343 (500 μg) for 72 hours (n= 5). The tumor tissues were isolated, and the proportion of DC, NK, and CD4T cells were analyzed by flow cytometry. Student *t*-test was performed to determine differences between groups, ^*^*P*< 0.05.


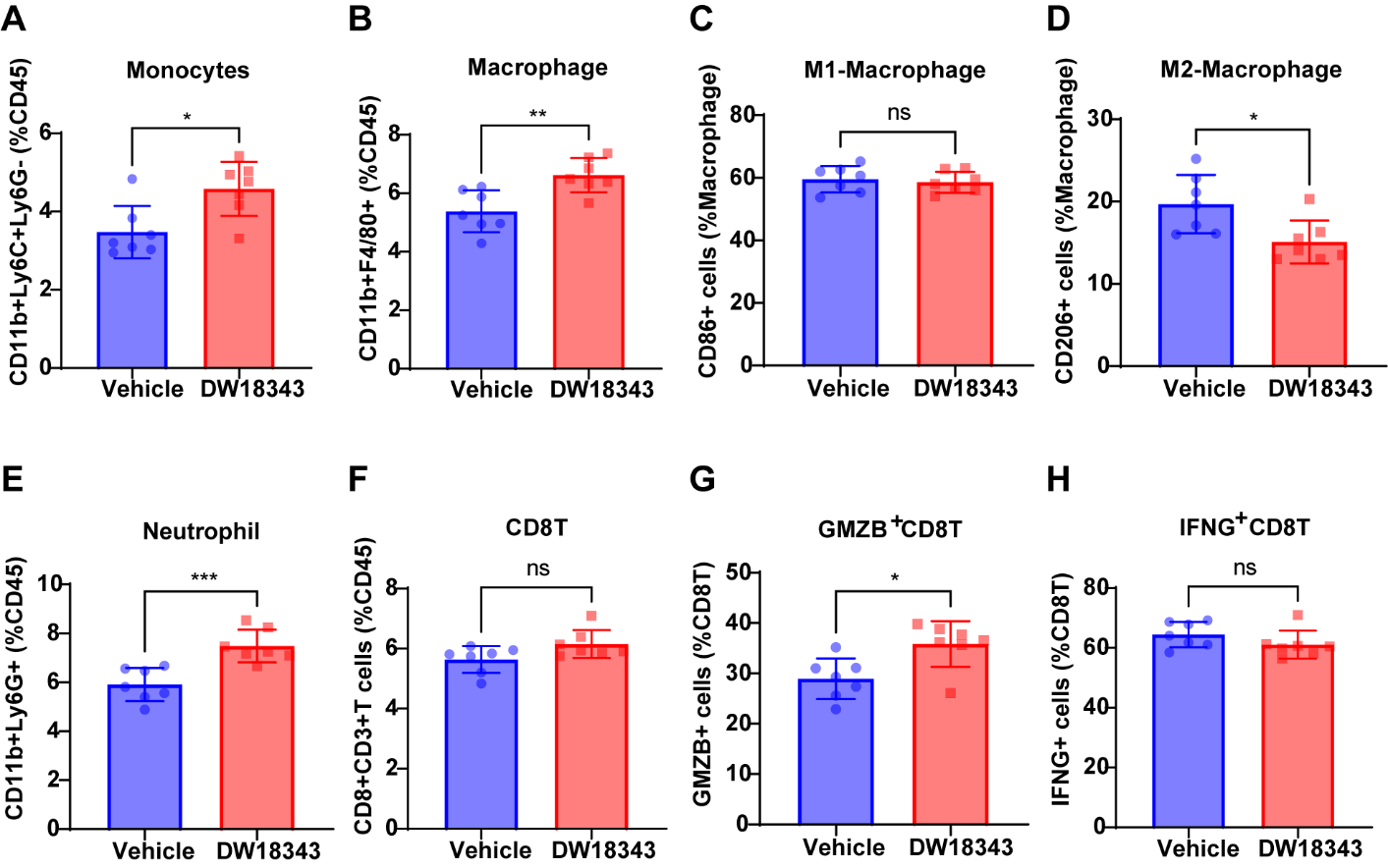


**Figure S6.** **Changes in peripheral blood immune cell subsets following treatment with DW18343.** (A-H) The immune cells from the peripheral blood of mice were treated with either a vehicle or DW18343 (10 μM) for 72 hours (n=7). The proportion of monocytes (A), macrophages (B), M1-Macrophages (C), M2-Macrophages (D), neutrophils (E), CD8T (F), GZMB^+^CD8T (G), and IFNG^+^CD8T (H) cells were analyzed by flow cytometry. Student *t*-test was performed to determine differences between groups; ns, no statistical difference, ^*^*P*< 0.05, ^**^*P*< 0.01, ^***^*P*< 0.001.
